# Tumor cell cycle regulation: integrated perspective of stage characteristics, regulatory networks, and signaling pathway intervention strategies

**DOI:** 10.1186/s43556-026-00411-w

**Published:** 2026-02-16

**Authors:** Qing Wan, Zhongmin Yang, Lian Huang, Yang Xia, Lihua Long, Zucai Xu, Jida Li

**Affiliations:** 1 Institute of Zoonosis, College of Public Health, Zunyi Medical University, Zunyi, Guizhou 563000 China; 2 Key Laboratory of Maternal and Child Health and Exposure Science, Guizhou Provincial Department of Education, Zunyi, Guizhou China; 3 College of Ocean Food and Biological Engineering, Jimei University, Xiamen, Fujian China; 4 Department of Neurology, Affiliated Hospital of Zunyi Medical University, Zunyi, Guizhou China; 5 Key Laboratory of Brain Function and Brain Disease Prevention and Treatment of Guizhou Province, Zunyi, Guizhou China; 6 Southwest Guizhou Vocational and Technical College, Xingyi, Guizhou China; 7 Guanling Autonomous County Disease Prevention and Control Center (Guanling Autonomous County Health Supervision Station), Anshun, Guizhou China

**Keywords:** Cell cycle dysregulation, Cell cycle regulatory network, Precision biomarkers, Targeted intervention strategies, Tumor therapy

## Abstract

Dysregulation of the cell cycle is one of the fundamental mechanisms underlying tumorigenesis, making cell cycle-related regulators potential antitumor therapeutic targets. Despite significant advances in understanding cell cycle regulatory networks, there is still a lack of a comprehensive and up-to-date synthesis that integrates the latest mechanistic insights with their translational potential in oncology. This review first systematically outlines the pivotal role of the cyclin-cyclin-dependent kinase (CDK)-cyclin-dependent kinase inhibitor (CKI) axis in driving aberrant cell cycle progression in tumors. Then the complex regulatory mechanisms of the tumor cell cycle were explored from various perspectives, including transcriptional control, post-translational modifications, checkpoint mechanisms, crosstalk with cellular processes, and integration with key signaling pathways. Furthermore, we highlight a series of clinically relevant biomarkers tightly linked to cell cycle dysregulation. Focusing on approved therapeutic agents and natural compounds in clinical trials, current treatment approaches that target the cell cycle and related metabolic pathways were also comprehensively assessed, and their prospects in precision oncology were elaborated. Finally, we discuss persistent challenges, including the incomplete understanding of tumor-specific cell cycle networks and the barriers to the clinical translation of targeted therapies. We advocate for future research to leverage multi-omics integration and systems biology approaches to facilitate more precise and effective cell cycle-directed interventions. This work offers a comprehensive framework that connects the fundamental mechanisms of cell cycle dysregulation in tumors with clinical translation, aiming to accelerate biomarker discovery and the development of next-generation precision oncology strategies.

## Introduction

The cell cycle is a tightly controlled, highly ordered series of biological processes that results in cellular proliferation by producing genetically identical daughter cells through mitosis [1]. This process is pivotal for maintaining tissue homeostasis and ensuring normal physiological functions of the organism, as it effectively restores cells lost to apoptosis or senescence. Classically, the cell cycle is divided into four consecutive phases, G1, S, G2, and M, each with unique functions and regulatory checkpoints [2]. In the G1 phase, cells grow larger, accumulate nutrients, and prepare for DNA replication [3]. The S phase completes the precise replication of DNA [4], converting the diploid content to tetraploid. G2 is a period that involves the synthesis of essential proteins to prepare the cell to go into mitosis [5]. Finally, the M phase ensures equitable chromosome segregation, yielding two genetically identical daughter cells [6]. Research demonstrates that the orderly progression of the cell cycle relies on a multi-level, dynamically coordinated regulatory network [7]. Among these, the complex formed by cyclins and CDKs constitutes the core engine driving the cell cycle process. Its activity is precisely regulated through multiple mechanisms, including inhibitory proteins (such as CDK inhibitors, CKIs) and phosphorylation modifications [8]. Additionally, cell-cycle checkpoints are established to monitor the process as critical quality-control gateways [9]. At pivotal transition points, such as G1/S and G2/M, they monitor parameters including DNA integrity, replication completion, and cell size, ensuring progression to the next phase only when conditions are fully favorable [10]. Meanwhile, the integration of transcriptional regulation [11], post-translational modifications [11], and extracellular signaling pathways [12] collectively constructs a highly dynamic, plastic, and precisely responsive cell cycle regulatory network capable of adapting to both internal and external environmental changes.

**Fig. 1:**
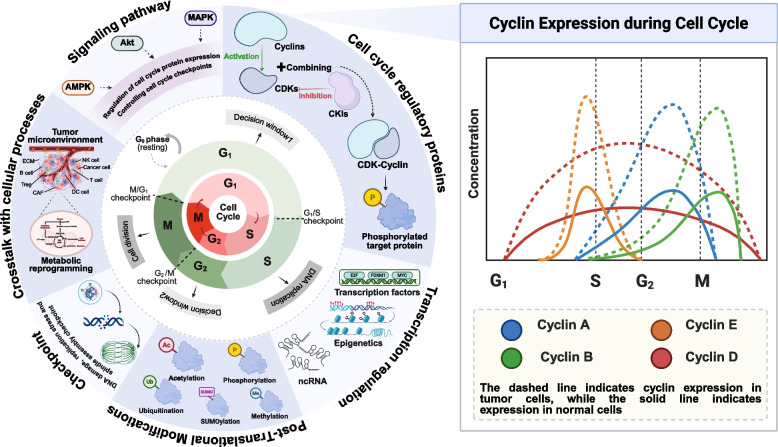
The cell cycle and its regulatory network. The inner ring illustrates the tumor cell cycle, the middle ring depicts the normal cell cycle, and the outer ring integrates the molecular network. The cell cycle progresses through G1 (preparation for DNA synthesis), S (DNA replication), G2 (preparation for mitosis), and M (mitosis). Tumor cells display accelerated cycling and elevated cyclin expression proteins compared to normal cells. Moreover, cell cycle progression is governed by a dynamic network involving cyclins, transcriptional regulation, post-translational modifications, checkpoint mechanisms, crosstalk with other cellular processes, and diverse signaling pathways. Abbreviations: AMPK, AMP-activated protein kinase; MAPK, mitogen-activated protein kinases; Akt, AKR mouse strain thymoma. (figure was created with Biorender.com)

However, tumorigenesis is closely associated with abnormalities in cell cycle regulation [13]. Compared with normal cells, tumor cells typically exhibit shortened cell cycle duration and accelerated proliferation rates, accompanied by significant upregulation of cyclin expression (such as Cyclin D/E) and their corresponding kinases (such as CDK4/6) (Fig. 1) [14–18]. It is worth noting that the latest research shows that tumor cells do not multiply completely uncontrollably. They selectively destroy key control points, such as cell cycle exit mechanisms, while preserving or even utilizing certain checkpoint functions to cope with replication stress and genomic instability. For instance, although DNA damage response pathways are frequently inactivated in tumors, the replication stress response and mitotic checkpoint often remain intact, acting as a lifeline for cancer cell survival [19, 20]. This vulnerability-dependent phenomenon provides a novel theoretical foundation for precision-targeted therapy. More importantly, the molecular mechanisms underlying tumor cell cycle dysregulation involve not only mutations or abnormal expression of core regulatory factors [21, 22], but also transcriptional imbalances [23], post-translational modification abnormalities [24], and functional defects in key cell cycle checkpoints [25]. These mechanisms are integrated within complex signaling networks and dynamically interact with cellular processes [25], collectively driving the abnormal proliferation, survival, and invasion of tumor cells (Fig. 1). This multi-layered regulatory system forms a sophisticated and dynamic network that not only determines the fate decisions of tumor cells, but also directly influences their response sensitivity to chemotherapy, targeted therapies, and immunotherapy.

Therefore, delving into the dynamic regulatory networks governing the tumor cell cycle and identifying its critical nodes and potential vulnerabilities is not only a core component in elucidating the mechanisms of tumorigenesis but also provides a pivotal foundation for theoretical innovation and therapeutic practice in precision oncology. In this review, we adopt an integrative perspective to systematically organize the characteristic molecular mechanisms, regulatory networks, and key biomarkers across all phases of the tumor cell cycle. We further explore intervention strategies targeting core cell cycle pathways. Through the development of comprehensive research, we aimed to establish a dynamic and systematic theoretical framework for enhancing our understanding of tumor cell cycle abnormalities. Thereby accelerating the conversion of fundamental research into clinical applications and aiding in the formulation and refinement of novel therapeutic strategies.

## Molecular machinery of the tumor cell cycle

**Fig. 2:**
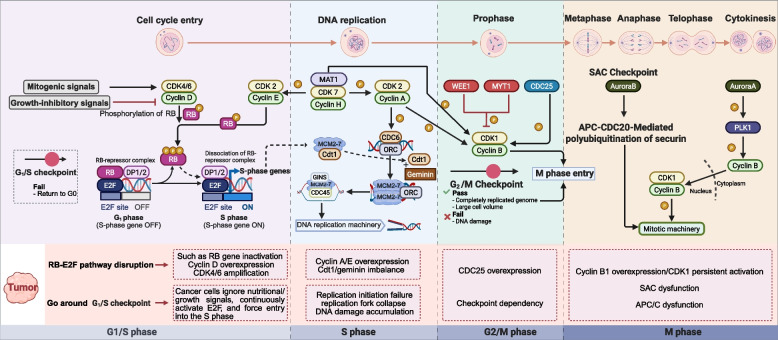
Regulatory mechanisms of the cell cycle. Cell cycle progression is driven by stage-specific Cyclin-CDK complexes and tightly controlled checkpoints. At the G1/S transition, sequential RB phosphorylation by Cyclin D-CDK4/6 and Cyclin E-CDK2 releases E2F to activate S-phase genes. During the S phase, the CDC45·MCM2-7·GINS helicase complex ensures faithful DNA replication, supported by CDK-activating kinases (e.g., Cyclin H-CDK7). The G2/M transition is governed by Cyclin B1-CDK1, whose activity is inhibited by WEE1/MYT1 and activated by CDC25. In mitosis, APC/CCDC20-mediated securin degradation triggers sister chromatid separation, while PLK1 and Aurora A regulate mitotic exit. Dysregulation of these pathways in cancer, such as cyclin overexpression, CDC25 upregulation, or spindle checkpoint defects, compromises genomic integrity and drives uncontrolled proliferation. Abbreviations: RB, retinoblastoma protein; DP1/2, dimerization partner 1/2; MAT1, menage a trois 1; MCM2-7, microchromosome maintenance 2–7; Cdt1, chromatin licensing factor 1; CDC45, cell division cycle 45; ORC, origin recognition complex; WEE1, Wee1 protein kinase-1; MYT1, myelin transcription factor 1; APC/C, anaphase-promoting complex; PLK1, polo-like kinase 1; Aurora A, Aurora kinase A. (figure was created with Biorender.com)

In normal cells, the cell cycle is precisely controlled by a series of Cyclin-CDK complexes, ensuring accurate DNA replication and faithful mitotic division while preserving genomic integrity. Cyclin D-CDK4/6 complexes initiate the cell cycle by retinoblastoma protein (RB), allowing the G1/S transition [26, 27]. Later, CDK2, paired with Cyclin A/E, further phosphorylates RB to ensure progression through S and G2 phases [28, 29]. Finally, the Cyclin B-CDK1 complex drives the cell to enter and progress through mitosis. In tumor cells, however, these regulatory mechanisms are often compromised, resulting in CDK hyperactivation, checkpoint bypass, and uncontrolled proliferation, which ultimately result in malignancy [30–34] (Fig. 2).

### G1/S phase: breakthrough and loss of control at limit points

The G1/S transition marks the restriction point (R point), a pivotal decision gate in the cell cycle that determines whether cells commit to DNA synthesis (Fig. 2) [35]. During the early G1 phase, cells integrate extracellular growth signals, nutrient availability, and intracellular stress cues to determine whether to proceed with proliferation, remain arrested, or exit into quiescence (G0) [36–40]. This decision is primarily governed by growth factor-dependent CDK activity, which is mainly mediated by complexes formed between D-type Cyclins (Cyclin D1, D2, and D3) and CDK4/6 [41–43]. Cyclin D binds to CDK4/6 and activates its kinase activity, irreversibly propelling cells from G0 into G1 [44–46]. Furthermore, for cells to enter the S phase, they must successfully cross the R point. When enough nutrients and energy have been accumulated and the cell has reached a sufficient size during the early G1, the originator gene will be activated, propelling the cell irreversibly through the R point into the late G1 phase [47, 48]. This model is supported by seminal work in yeast by Adler et al. [49], which revealed that cells remain responsive to environmental cues before the R point but are irreversibly committed to DNA replication once it is passed. Thus, the R point serves as a decisive boundary between cycle entry and exit, with the Cyclin D-CDK4/6 complex acting as a central molecular regulator of this transition.

As a primary repressor of E2F transcription factor (E2F), RB plays a central regulatory role in cell cycle progression decisions (Fig. 2) [50–53]. During the G1 phase, before DNA replication begins, unphosphorylated RB tightly binds to E2F1/2/3 transcription factors, inhibiting E2F-dependent transcriptional activity and thereby arresting cells in G1 [54, 55]. When growth factor signals are activated, the Cyclin D-CDK4/6 complex initiates RB phosphorylation, leading to partial release of E2F [56–58]. The released E2F then forms a heterodimer with dimerization partner 1/2 (DP1/2), triggers the transcription of downstream target genes, such as Cyclin E and other proteins and enzymes necessary for DNA synthesis, thereby driving the G1/S transition. It is worth noting that while the initial phosphorylation of RB by Cyclin D-CDK4/6-mediated (manifesting as monophosphorylation or partial phosphorylation) can induce partial release of E2F and initiate early gene expression [59–61]. Recent findings indicate that this process may be insufficient to fully activate E2F-dependent transcription and drive cells irreversibly into the cell cycle [62]. Instead, it positions cells in a primed state, preventing them from exiting the G1 phase. Furthermore, Cyclin E-CDK2 further hyperphosphorylates RB, completely suppressing its activity, enhancing E2F transcriptional activity, and forming a positive feedback loop. This ultimately facilitates cells to enter the G1/S transition (a crucial point of commitment in the cell cycle) [63]. Although Cyclin D-CDK4/6 may not directly cause the complete activation of E2F, it plays an indispensable role in the process of RB hyperphosphorylation [64]. Building upon this, reports indicate that Cyclin D-CDK4/6 can specifically recognize an α-helical structure at the C-terminal end of the RB protein and initiate its phosphorylation [65]. However, this particular site is not identifiable by Cyclin A/B/E. If this helix undergoes mutation, RB cannot be effectively phosphorylated, leading to cell cycle arrest at the G1 phase [66]. These findings imply that, in some cellular contexts, Cyclin E-CDK2’s function may be impaired or even insignificant, highlighting the significant cell-type dependence of the G1/S regulatory mechanism. Consequently, the synergistic regulation of RB phosphorylation is a crucial cornerstone of scientific investigations, as this process significantly participates in driving the cell’s decision to commit to cell division.

Cancer cells hijack the key regulatory mechanisms of the G1/S transition to proliferate uncontrollably during tumorigenesis. They primarily disrupt the core function of the RB-E2F pathway, enabling evasion of the restriction point checkpoint and bypassing the critical decision window in the early G1 phase. This forces cells irreversibly into S phase. While such dysregulation of the tightly controlled cell cycle drives malignant progression, it also creates a therapeutic opportunity.

### S phase: DNA replication and quality control

The S phase (DNA synthesis phase) is a vital stage in the cell cycle characterized by high-fidelity genome replication and the coordinated production of chromosome-associated components (like centrosomes and histones) [67]. This process is highly dependent on two core regulatory axes, namely CDK7-Cyclin A-CDK2 and E2F-Rb. These together ensure the precise segregation of chromosomes during the ensuing mitotic division.

The Cyclin A-CDK2 complex serves as a central regulator of S-phase progression (Fig. 2). According to research, this complex controls the function of cell division cycle 6 (CDC6) in the initiation of DNA replication through phosphorylation, which causes CDC6 to translocate from the nucleus in the G1 phase to the cytoplasm [68]. This mechanism effectively prevents DNA replication during the S phase and the G2 phase. Furthermore, Cyclin A-CDK2-mediated phosphorylation promotes the degradation of chromatin licensing factor 1 (Cdt1). CDC6 translocation, Cdt1 degradation, and geminin work together to prevent the minichromosome maintenance protein complex (MCM) from reassociating with chromatin during S phase [69], thereby preventing the S-G2 transition. Further studies reveal that activation of the Cyclin A-CDK2 complex is tightly regulated by the CDK-activating kinase (CAK), composed of Cyclin H, CDK7, and myelin transcription factor 1 [70]. CAK activates CDK1/2 by phosphorylating a conserved threonine residue in the T-loop (for example, Thr160 in CDK2), conferring full kinase activity and enabling orderly S-phase progression [71]. Thus, inhibition of CDK7 disrupts Cyclin A-CDK2 activation, impairs DNA replication initiation, and induces S-phase arrest.

S-phase progression is also critically dependent on sustained RB phosphorylation and E2F activation. Continuous inactivation of RB ensures sufficient release of E2F transcription factors, which drive the expression of Cyclin A/E, thereby maintaining CDK2 activity throughout the S phase [72, 73]. Once the S phase is completed, Cyclin A/B binds to CDK1, causing cells to enter mitosis [74]. Conversely, impaired Rb phosphorylation suppresses E2F activity, resulting in downregulated Cyclin A/E transcription and diminished CDK2 activity. This disrupts Cyclin A/E-CDK2 complex formation, ultimately triggering S-phase arrest. These findings highlight the E2F-Rb-CDK2 axis as a potential therapeutic target. Furthermore, DNA replication initiation depends on the sequential assembly of multiple pre-replicative complexes (pre-RCs). The complex formed by the origin recognition complex (ORC), CDC6, and Cdt1 recruits the MCM2-7 hexamer onto replication origins, creating a closed double-ring structure [75]. As cells enter the S-phase, CDC45 and the GINS complex associate with MCM2-7 to form the CMG (CDC45·MCM2-7·GINS) helicase [76]. Activated by phosphorylation from CDK and DDK, the CMG complex unwinds the DNA double helix and facilitates the replication fork progression (Fig. 2).

In tumor cells, S-phase is often accompanied by uncontrolled replication initiation, impaired replication fork stability, and increased replication stress, leading to genomic instability that promotes malignant progression [77–79]. Targeting key factors such as CDK7 or CDK2 can disrupt Cyclin A-CDK2 activation or weaken E2F-driven transcription [80]. This action impairs pre-RC assembly and CMG helicase activation, selectively inhibiting tumor cell proliferation.

### G2/M phase: the switch that initiates mitosis

The G2 phase is a pivotal interval in the cell cycle, during which the cell prepares for mitosis following DNA replication. This period involves cell growth, synthesis of proteins and organelles needed for division, repair of DNA damage, and accumulation of energy reserves [81]. In addition, microtubule proteins undergo structural reorganization, establishing the foundational framework for mitotic spindle assembly [82]. At the end of the G2 phase, the G2/M checkpoint ensures that DNA replication is complete and genomic integrity is preserved before mitotic entry [81]. Cells encountering DNA damage or incomplete replication activate the ataxia telangiectasia and rad3-related protein-checkpoint kinase 1 (ATR-CHK1) signaling pathway, which activates CDK1 and prevents premature mitotic entry [83]. This checkpoint mechanism is essential for maintaining genomic stability and restricting the propagation of damaged genomes.

Following successful passage through the G2/M checkpoint, cells initiate the transition to M phase. The core regulatory mechanism of this process relies on the complex formed by Cyclin B and CDK1 (known as the maturation-promoting factor, MPF) (Fig. 2) [84]. Cyclin B (primarily Cyclin B1 and B2) steadily accumulates beginning in the S phase under cell cycle-specific transcriptional regulation. It then forms a complex with CDK1 when the optimum concentration is achieved [85]. However, Wee1 protein kinase-1 (WEE1) and membrane-associated tyrosine and threonine-specific myelin transcription factor 1 (MYT1), which are responsible for phosphorylating CDK1 subunits at threonine-14 and threonine-15, initially keep this complex inactivated. MPF activation requires dephosphorylation of these sites by CDC25 phosphatases (Fig. 2) [86]. Thus, WEE1/MYT1 and CDC25 maintain a dynamic balance. This balance acts as the key switch that controls CDK1 activation. Moreover, the balance is tightly controlled by multiple feedback loops. When activated, CDK1 further activates CDC25 and inhibits WEE1, forming a positive feedback loop that quickly enhances MPF activity, facilitating a rapid and irreversible bistable transition from G2 to M phase [87–89]. Additionally, kinases such as glycogen synthase kinase-3 β (GSK-3β) indirectly influence the phosphorylation of CDK1 substrates by modulating the activity of associated phosphatases. This further contributes to the stability of the regulatory network.

Tumor cells often overexpress CDC25, which disrupts the G2/M checkpoint and permits mitotic entry despite DNA damage [90]. Conversely, certain tumor types exhibit checkpoint dependency, relying on WEE1 or CHK1 to sustain G2 arrest and facilitate DNA repair [91, 92]. This vulnerability can be exploited for the development of efficacious cancer treatments that target WEE1 or CHK1. Although Cyclin B accumulates steadily during the G2/M phase, this accumulation is not enough to directly initiate mitosis. Rather, the commitment to mitosis is accomplished through rapid, switch-like activation of CDK1, governed by the dynamic interplay among WEE1, MYT1, and CDC25, coupled with a CDK1-driven positive feedback loop. This circuitry generates a sharp, irreversible bistable transition, ensuring precise and timely mitotic onset [93]. Any disruption to the key elements of this tightly regulated system restricts MPF activation, prevents G2/M progression, and causes cell cycle arrest.

### M phase: the switch that initiates mitosis

The M phase is the final stage of the cell cycle that produces two genetically identical daughter cells. Hallmark events of mitosis include chromosome condensation, spindle assembly, chromosome alignment and separation, and cytokinesis. These processes are governed by highly conserved molecular mechanisms, among which the spindle assembly checkpoint (SAC) serves as the central surveillance system ensuring accurate chromosome segregation and maintaining genomic stability [94–99].

In mammalian cells, the Cyclin B-CDK1 complex functions as the master regulator of mitosis entry and progression (Fig. 2) [100, 101]. The Cyclin B family consists of three isoforms, namely Cyclin B1, B2, and B3. Cyclin B1 and B2 bind CDK1 to form an active complex that triggers the cell to go through mitosis [102], whereas Cyclin B3 complexes with CDK2 to regulate mitosis in specific cell types or during specific biological processes [103–105]. The optimal activation of CDK1 triggers a cascade of events leading to mitosis, induced by the phosphorylation of diverse substrate proteins. CDK1 further activates multiple key mitotic kinases, such as polo-like kinase 1 (PLK1), Aurora kinase A, and Aurora kinase B. These kinases synergistically regulate centrosome maturation, spindle assembly, kinetochore-microtubule attachment, and SAC signaling [106, 107].

During the prophase stage of mitosis, increased CDK1 activity induces morphological cellular changes and centrosome separation [108]. It triggers nuclear membrane rupture by phosphorylating nucleolar proteins, which exposes chromatin to the cytoplasmic microtubule environment, consequently facilitating spindle assembly [109]. Additionally, enhanced nuclear import of Cyclin B1 drives chromosome condensation and nucleolar disassembly, laying the groundwork for later activation of the anaphase-promoting complex (APC/C) [110]. Chromosomes attach to spindle microtubules via kinetochores, and the SAC monitors proper bipolar attachment. Following this, the APC/C binds its co-activator CDC20, forming APC/CCDC20 and triggering mitotic exit [111, 112]. APC/CCDC20 first mediates the ubiquitin-mediated degradation of Cyclin A, releasing its inhibitory effect on the APC/C [113]. It then targets Cyclin B1/B2 for polyubiquitination, facilitating their degradation by the 26S proteasome [114]. CDK1 inactivation permits the reactivation of protein phosphatases, such as protein phosphatase 2A (PP2A)-B55, which systematically dephosphorylate CDK1 substrates and reverse structural rearrangements [115, 116]. This coordinated dephosphorylation drives sister chromatid separation, spindle elongation, contraction ring formation, and cytokinesis, ultimately completing cell division (Fig. 2) [117]. Tumor cells often disrupt M-phase regulation. They dysregulate SAC components (such as budding uninhibited by benzimidazoles 1, BUB1 and mitotic arrest deficient 2 (MAD2), cause chromosomal instability and aneuploidy. Furthermore, the overexpression of Cyclin B1 or prolonged CDK1 activation can be observed, as well as impaired APC/C function or excessive CDC20 activation, which also disturb mitotic timing and cause aneuploidy [118].

Mitotic exit is a rapid, coordinated, and irreversible process that relies on APC/CCDC20-mediated Cyclin B degradation and the feedback activation of protein phosphatases. Dysregulation of this mitotic regulatory network in cancer cells creates therapeutic vulnerabilities. Small-molecule inhibitors of CDK1, PLK1, Aurora kinases, and the APC/C pathway now guide anti-tumor drug efforts.

## The intricate regulatory networks governing the cell cycle

### Transcriptional regulation

**Fig. 3:**
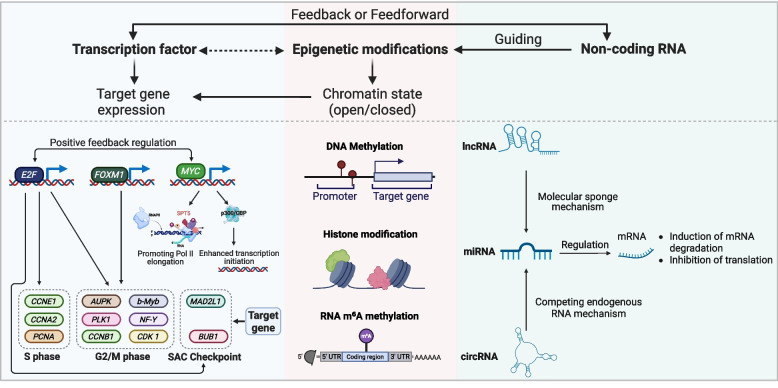
Mechanisms of transcriptional regulatory networks in cell cycle progression. Transcription factors (such as *E2F*, *FOXM1*, and *MYC*), epigenetic modifications (DNA methylation, histone modifications, and RNA m⁶A modification), and non-coding RNAs (lncRNAs, circRNAs and miRNAs) form an integrated network that dynamically controls the timing and fidelity of cell cycle gene expression. This ensures proper checkpoint function and genomic stability. Dysregulation of this network disrupts transcriptional homeostasis and contributes to tumorigenesis. Abbreviations: *E2F, E2F transcription factor; CCNE1, Cyclin E1; PCNA, proliferating cell nuclear antigen; FOXM1, forkhead box protein M1; AURK, Aurora kinases; PLK1, polo-like kinase 1; b-Myb, brain-type Myb; NF-Y, nuclear factor Y; CDK1, cyclin-dependent kinase 1; MYC, MYC proto-oncogene; BUB1, budding uninhibited by benzimidazoles 1; MAD2L1, mitotic arrest deficient 2 like 1.* (figure was created with Biorender.com)

The orderly progression of the cell cycle relies on a highly coordinated transcriptional regulatory network. This network encompasses the transcription factors, epigenetic modifications, and non-coding RNA (ncRNA) mechanisms. Together, they form a multilayered, active system that controls gene expression (Fig. 3) [119–121]. Under physiological conditions, this network sustains the balance between cell proliferation and quiescence. In tumorigenesis, however, its dysregulation triggers uncontrolled proliferation and chromosomal instability (CIN) [122–126].

The E2F-RB pathway acts as a central regulatory hub for the G1/S transition [127]. E2F transcription factors are crucial regulators controlling DNA replication and G2/M progression by modulating the expression of necessary S-phase genes (such as *CCNA2*, *CCNE1*, and *proliferating cell nuclear antigen*) and mitotic checkpoint genes (like *mitotic arrest deficient 2 like 1*, and *BUB1*), in synergy with co-factors such as *myb proto-oncogene like 2* and *nuclear factor Y (NF-Y)*. This pathway is frequently disrupted in human cancers through mechanisms that include RB1 inactivation (prevalent in retinoblastoma, osteosarcoma, and triple-negative breast cancer), Cyclin D1 amplification, or CDK4 mutations (commonly observed in breast cancer and mantle cell lymphoma) [128]. These changes cause Rb to become hyperphosphorylated, which releases E2F and causes uncontrolled S-phase gene transcription. As a key transcription regulator during the G2/M phase, *FOXM1* controls target genes such as *CCNB1, CDK1, PLK1, and Aurora kinases*, participating in processes including spindle assembly, centrosome maturation, and chromosome separation [129, 130]. When found highly expressed in cancers like hepatocellular carcinoma and breast cancer, the outcomes are usually poor, with potential tumor invasiveness [131]. Additionally, *MYC* recruits co-activators such as histone acetyltransferase p300/CBP to remodel chromatin structure and enhance transcription initiation. Furthermore, it promotes effective RNA polymerase II (Pol II) elongation by interacting with transcription elongation factors such as transcription elongation factor SPT5 [132]. Abnormal activation of *MYC* is widely observed in over 70% of human cancers [133]. Typical examples include *c-MYC* overexpression caused by the t(8; 14)(q24; q32) chromosomal translocation in Burkitt lymphoma (affecting approximately 80% of patients) [134, 135] and *N-MYC* gene amplification in high-risk neuroblastoma [136]. Amplification of transcriptional regulators such as CDK8/19 further potentiates *MYC*-dependent transcription in tumors, including colorectal cancer [137].

Epigenetic mechanisms dynamically regulate the expression of cell cycle genes through DNA methylation, histone modifications, and RNA m⁶A methylation [138–144]. To sustain transcriptional activity during the S/M phase transition, cells must accurately reset epigenetic marks (such as histone H3 trimethylated at lysine 4, H3K4me3) on new chromatin. Hypermethylation of CpG islands in promoters of genes like Cyclin D1 impedes G1/S progression, whereas aberrant methylation of tumor suppressor promoters (such as breast cancer type 1 susceptibility protein, BRCA1 and cyclin-dependent kinase inhibitor 2A, CDKN2A) compromises cyclin-dependent checkpoint integrity [145]. For histone modifications, G1/S progression is driven by H3K4me3 enrichment at Cyclin D1 and Cyclin E1 promoters. However, the depletion of CpG-binding protein lowers H3K4me3 levels and slows down cell proliferation. Coactivator-associated arginine methyltransferase 1 orchestrates transcription complex assembly by catalyzing H3R17me2a and methylating non-histone substrates; its interplay with the nucleosome remodelling and deacetylase complex is pivotal for G1/S control in breast cancer. In parallel, m⁶A RNA modification has emerged as an important post-transcriptional regulator of cell cycle dynamics, influencing RNA stability, translation, and precise chromosome segregation fidelity [146–152].

Additionally, ncRNAs further refine transcriptional output by modulating mRNA stability, translation, and protein activity [153]. MicroRNAs (miRNAs) induce degradation or translational repression by binding to target mRNAs [154]. For example, oncogenic miRNAs (such as miR-21 and miR-222) can enhance G1/S transition by activating pro-proliferative signaling pathways, whereas tumor-suppressive miRNAs (such as miR-34c-5p) induce cell-cycle arrest through cyclin inhibition [155, 156]. Long non-coding RNAs (lncRNAs) often function as molecular sponges that bind to and sequester miRNAs. By doing this, ncRNAs prevent miRNAs from repressing the expression of their targets. For instance, lncRNA HNF1A-AS1 binds miR-661 in gastric cancer, upregulating CDC34 to promote p21 ubiquitination and degradation, thereby bypassing G1 arrest and accelerating cell cycle progression [157]. Owing to their covalently closed-loop structure, Circular RNAs (circRNAs) exhibit exceptional stability and frequently serve as competitive endogenous RNAs [158–160]. In acute myeloid leukemia, circRNA RP11-641D5.1 binds to miR-486-5p, releasing its inhibition of Cyclin D1 and promoting cell proliferation. In gastric adenocarcinoma, circRNA_0007766 competitively binds to miR-34c-5p, upregulating Cyclin D1 expression and driving tumor growth.

Under physiological conditions, transcription factors, epigenetic modifications, and non-coding RNAs form a highly synergistic regulatory axis to precisely manage the cell cycle and its checkpoints. Transcription factors regulate the expression of cycle-related genes by directly binding to target gene promoters. Epigenetic mechanisms dynamically modulate chromatin states [161]. Non-coding RNAs function as bridge molecules, guiding the positioning of epigenetic complexes through base pairing or protein interactions and forming feedback loops with transcription factors to collaboratively reshape the transcriptional landscape. However, this network often experiences pathological reconfiguration in tumor microenvironments, resulting in cycle dysregulation [162]. Recent advances in CRISPR-dCas9-mediated targeted epigenetic editing, along with ncRNA-targeted therapies [163], provide precise interventions that preserve sequences within this co-regulatory axis, signaling a transformative potential for therapy.

### Post-translational regulation

**Fig. 4:**
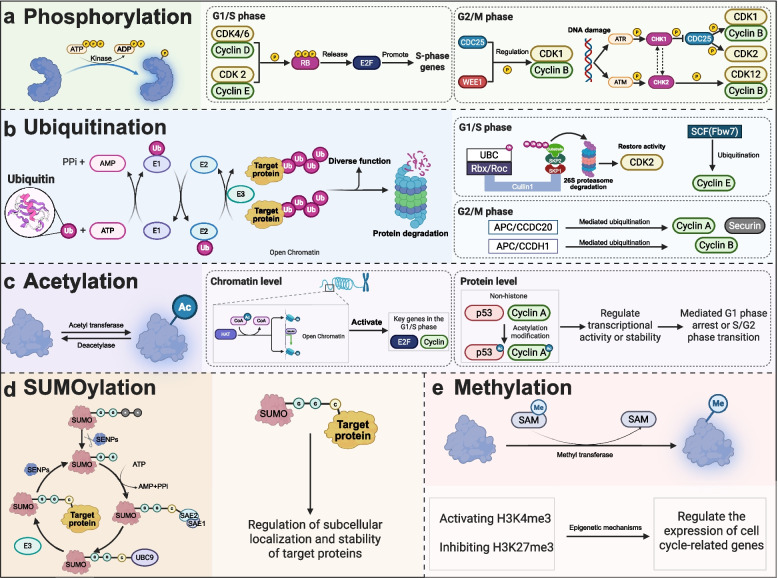
Mechanisms of post-translational protein modifications in cell cycle regulation. **a** Phosphorylation regulation: Phosphorylation modifications of proteins such as Rb, Cyclin-CDK complexes, CDKs, and CHKs play crucial regulatory roles in the transition from the G1/S phase to the G2/M phase. **b** Ubiquitination regulation: The SCF E3 ubiquitin ligase complex facilitates the G1/S transition and initiates DNA replication by mediating the ubiquitin-mediated degradation of cell cycle inhibitors, such as p27Kip1. Subsequently, the APC/C complex triggers sister chromatid separation and mitotic exit through degradation of Cyclin B and securin. **c** Acetylation regulation: Histone acetylation enhances chromatin accessibility to activate transcription of cell cycle-related genes. Additionally, non-histone acetylation modulates transcriptional activity, protein stability, or subcellular localization. **d** SUMOylation regulation: Covalent attachment of SUMO moieties regulates the subcellular localization and stability of critical cell cycle factors, enabling fine-tuned control of their functions. **e** Methylation regulation: Methylation regulates the expression of cell cycle-related genes through epigenetic mechanisms. Abbreviations: RB, retinoblastoma protein; WEE1, Wee1 protein kinase-1; CDC25, cell division cycle 25; ATR, ataxia telangiectasia mutated and rad3-related protein; ATM, ataxia telangiectasia mutated; CHK1, checkpoint kinase 1; SCF, Skp1-Cullin1-F-box ubiquitin ligase complex. (figure was created with Biorender.com)

Proteins are central effectors of cell cycle control, and their activity is precisely modulated by post-translational modifications (PTMs), including phosphorylation, ubiquitination, acetylation, and sumoylation to dynamically regulate their conformation [164–167]. These covalent modifications dynamically modulate protein conformation, stability, subcellular localization, and interaction networks, thereby enabling accurate execution of cell-cycle transitions (Fig. 4) [168]. While PTMs maintain checkpoint fidelity and homeostasis in normal cells, their dysregulation in cancer promotes uncontrolled proliferation, genomic instability, and tumor progression.

Phosphorylation is the primary PTM governing cell cycle progression through reversible regulation of Cyclin-CDK activity. During the G1/S transition, sequential phosphorylation of RB by Cyclin D-CDK4/6 and Cyclin E-CDK2 complexes releases the E2F transcription factor, initiating activated S-phase gene expression [169]. During G2/M, Cyclin B-CDK1 activity is tightly controlled by the opposing actions of Wee1 kinase and CDC25 phosphatase. In response to DNA damage, ATM/ATR-mediated activation of CHK1/CHK2 inhibits CDC25, suppresses CDK1/2 activity, and enforces cell cycle arrest to facilitate DNA repair [170]. In malignancies, aberrant CDK signaling frequently disrupts phosphorylation homeostasis. In gastric cancer and pancreatic cancer, CDK2-mediated phosphorylation suppresses sirtuin 5, causing the Warburg effect and proliferation [171, 172]. In colorectal cancer, CDK8 phosphorylates Yes-associated protein 1, which promotes migration and invasion [156]. In breast and lung cancers, Cyclin D1-CDK4/6 phosphorylation stabilizes glutathione S-transferase Pi 1, accelerating mitosis and precipitating cell cycle dysregulation [173, 174].

Ubiquitination acts as a molecular timer that ensures unidirectional and irreversible cell cycle progression by controlling the timely degradation of key regulators [175, 176]. At the G1/S phase, the Skp1-Cullin1-F-box ubiquitin ligase complex (SCF) specifically recognizes and ubiquitinates the cell cycle inhibitor p27Kip1 through its substrate recognition subunit S-phase kinase-associated protein 2 [177, 178]. This facilitates its degradation through the 26S proteasome pathway, relieving inhibition of CDK2 and promoting S-phase entry [179]. Conversely, SCFFbw7 mediates Cyclin E’s ubiquitinylation to facilitate its prompt degradation and avoid excessive CDK2 activation. Cyclin A, a CDK1 regulator, and securin are ubiquitinated when APC/C and the coactivator CDC20 form APC/CCDC20 during G2/M entry [180]. Securin degradation activates separase, triggering sister chromatid separation [181]. During the metaphase-to-telophase transition, APC/C switches partners to cadherin 1 (CDH1), forming APC/CCDH1, which selectively ubiquitinates Cyclin B [182]. By silencing CDK1 activity, mitotic exit is facilitated, which ensures the proper progression of the cell cycle [183]. In cancers, dysregulated ubiquitination contributes to immune evasion, epithelial-mesenchymal transition, metabolic rewiring, and oncogenic signaling. For example, the deubiquitinase ubiquitin-specific peptidase (USP22) stabilizes programmed cell death ligand 1 (PD-L1) across multiple solid tumors, fostering immune suppression and therapy resistance [184]. In gastric cancer, USP38 stabilizes fatty acid synthase (FASN), enhancing glycolysis and metabolic adaptation [185]. In lung cancer, the mouse double minute 2 homolog and tripartite motif-containing 28 interact to ubiquitinate and degrade p53, triggering CDK activity and contributing to cell cycle dysregulation [186].

Acetylation regulates cell cycle progression through its influence on gene expression and protein stability, coordinated by acetyltransferases and deacetylases in chromatin remodeling and non-histone functions [187]. At the chromatin level, histone acetylation, such as H3K27ac, relaxes chromatin structure and facilitates transcription of genes required for G1/S and G2/M transition genes [188, 189]. At the protein level, acetylation of non-histones directly regulates their transcriptional activity or stability, thereby mediating G1 arrest or S/G2 transition [190]. This forms a multi-tiered regulatory network ensuring precise cell cycle execution. For example, high mobility group nucleosome-binding protein 2 promotes G2/M transition in glioblastoma by stabilizing H3K27ac at the CDC20 promoter to enhance its transcription [191]. Similarly, lysine acetyltransferase 8 drives cell cycle progression across multiple tumors by regulating H4K16ac levels [192].

Additional PTMs contribute to fine-tuning cell cycle regulation. SUMOylation modulates the subcellular localization and stability of target proteins via covalent conjugation of SUMO moieties [193–196]. For example, SUMOylation of p21 enforces its nuclear retention, inducing G1-phase arrest [197], whereas SUMOylation of E2F suppresses its activity, preventing aberrant S-phase entry [55]. Methylation epigenetically governs the expression of cyclin-associated genes through various mechanisms involving histone modifications (like H3K4me3 marks and H3K27me3 marks) [198].

Importantly, PTMs operate not in isolation but as an integrated code. Phosphorylation-dependent phosphodegrons recruit ubiquitin ligases, acetylation can compete with ubiquitination or SUMOylation at shared lysine residues, and NAD(+)-dependent deacetylases link cellular metabolic status to cell cycle dynamics. Disruption of this interconnected PTM network is a defining feature of tumor cell cycle dysregulation. Elucidating PTM crosstalk and context-specific dependencies will be essential for developing effective therapeutic strategies targeting post-translational control of the cell cycle.

### Cell cycle checkpoints

**Fig. 5:**
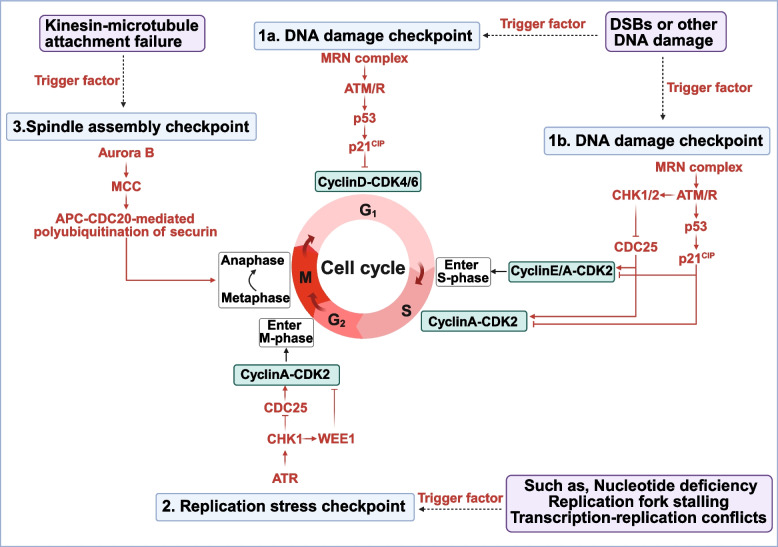
Regulatory mechanisms of core cell cycle checkpoints. The DNA damage checkpoint is activated by ATM/ATR, leading to p53-dependent p21 induction and CHK1/2-mediated inhibition of CDC25, collectively blocking Cyclin-CDK activity and arresting cells in G1. The replication stress checkpoint relies on ATR-CHK1 signaling, which sustains inhibitory phosphorylation of CDKs via WEE1 activation and CDC25 suppression, thereby slowing replication or delaying mitotic entry. The spindle assembly checkpoint prevents anaphase onset until all kinetochores are properly attached; unattached kinetochores trigger Aurora B-dependent formation of the MCC, which inhibits APC/CCDC20 and stabilizes securin and Cyclin B to block sister chromatid separation. Abbreviations: ATR, ataxia telangiectasia mutated and rad3-related protein; ATM, ataxia telangiectasia mutated; CHK1/2, checkpoint kinase 1/2; WEE1, Wee1 protein kinase-1; CDC25, cell division cycle 25; MCC, mitotic checkpoint complex. (figure was created with Biorender.com)

Cell cycle checkpoints are evolutionarily conserved mechanisms that prevent cell cycle progression in response to signals like DNA damage, DNA replication stress, or abnormal spindle assembly. They work by inhibiting CDK activity and activating the APC/C, which arrests or slows the cell cycle to allow for repair or apoptosis (Fig. 5) [199].

#### DNA damage checkpoints

DNA damage checkpoints detect injuries such as double-strand breaks (DSBs) through the ATM-CHK2 and ATR-CHK1 signaling pathways [25]. Upon DNA damage, the MRN complex activates ATM, which phosphorylates downstream effectors such as CHK2 and p53, triggering cell cycle arrest, DNA repair, or apoptosis to protect genomic integrity [200]. In the G1 phase, the p53/p21 axis inhibits CDK2 expression, thus blocking G1/S progression [201]. During the S/G2 transition, CHK2 promotes CDC25 degradation and WEE1 activation, consequently suppressing CDK1 and halting mitosis [202]. Additionally, ssDNA arising from DSB resection engages the ATR-CHK1 pathway, reinforcing S/G2 arrest [203]. Loss of p53 and ATM function is central to DNA damage checkpoint dysregulation [204]. Mutations in p53 corrupt the G1 arrest in cells, making cells reliant on the S/G2 checkpoint and promoting adaptive tolerance. DSB repair is diverted to an error-prone non-homologous end joining due to ATM loss, which results in chromosomal rearrangements. Despite S/G2 redundancy, HPV E6/E7 proteins degrade p53 and RB, corrupting both G1/S and S/G2 checkpoints and amplifying genomic instability [205].

#### Replication stress checkpoint

The replication stress checkpoint monitors DNA replication during the S phase and is predominantly governed by the ATR-CHK1 pathway [206]. Aberrant forks are indicated by the accumulation of ssDNA that occurs when the DNA helicase and polymerase activities become uncoupled [207]. Causes include nucleotide depletion, topological constraints, transcription-replication collisions, and exogenous insults such as topoisomerase inhibitors. When the pathway is activated, CHK1 facilitates the degradation of CDC25 and the upregulation of WEE1, inducing the inhibition of CDK1/2 through tyrosine 15 phosphorylation. This ultimately leads to G2/M arrest [208]. Concurrently, it suppresses late-origin firing and stabilizes stalled forks, averting their collapse into double-strand breaks. This “pause-and-repair” strategy ensures mitosis proceeds only after replication completion and damage resolution [209]. In tumors, chronic replication stress induced by oncogene overexpression (such as *MYC*) renders cancer cells highly dependent on the ATR-CHK1 pathway for maintaining genomic stability [210]. ATR deficiency exacerbates replication fork collapse, and in BRCA-deficient tumors, inhibition of either ATR or CHK1 produces a synthetic lethal effect [211]. Clinically, CHK1 overexpression in triple-negative breast cancer correlates strongly with chemoresistance, underscoring the ATR-CHK1 axis as a prime therapeutic target [212].

#### Spindle assembly checkpoint

SAC safeguards chromosome segregation during mitosis by delaying anaphase onset until all chromosomes achieve proper bipolar attachment to spindle microtubules [213–215]. The mitotic checkpoint complex (MCC), composed of BUB3, MAD2, BUBR1, and CDC20, is formed by this signal and effectively inhibits the APC/CCDC20 ubiquitin ligase [216, 217]. By blocking the degradation of substrates such as Cyclin B, the MCC sustains CDK1 activity and preserves chromosomal fidelity [218]. SAC dysfunction (like MAD2 overexpression or BUB1 mitotic checkpoint serine/threonine kinase B (BUB1B) loss) triggers premature APC/C activation, leading to chromosome missegregation and aneuploidy [219]. This drives CIN, promoting tumor heterogeneity and metastasis. For example, BUB1B deficiency in colorectal cancer markedly amplifies CIN and accelerates cancer progression [220]. Notably, many cancer cells maintain a degree of SAC functionality and rely on it for their survival, creating selective vulnerabilities that can be exploited, while normal cells exhibit greater checkpoint redundancy.

#### Checkpoint crosstalk and therapeutic implications

Cell cycle checkpoints exhibit a double-edged sword characteristic in tumors, where their dysfunction drives malignant transformation while simultaneously presenting critical therapeutic windows. Extensive crosstalk exists between pathways such as DNA damage response, replication stress regulation, and spindle assembly monitoring. For example, DNA damage induces replication stress, and ATR-CHK1 activation intensifies S/G2 arrest, while the ATM-CHK2-p53 pathway reinforces the G1/S barrier [221]. The function of the spindle checkpoint also relies on upstream pathway support [221]. Notably, this interaction not only endows the checkpoint system with functional redundancy but also reveals specific vulnerabilities in tumor cells. Cells in tumor models lacking p53 or ATM are highly dependent on G2/M and spindle checkpoints, and replication stress caused by oncogenes such as *MYC* aggravates multi-pathway synergistic dependency. Future therapeutic strategies should leverage the cross-regulatory properties of checkpoints to develop multi-target inhibitors inducing synthetic lethality. Combining tumor genetic backgrounds (such as BRCA defects) with checkpoint activity biomarkers may enable precision interventions.

### Crosstalk with cellular processes

**Fig. 6:**
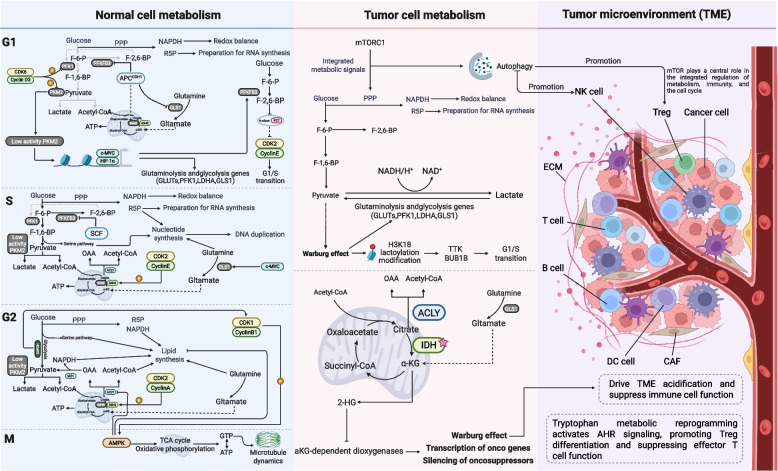
Cell cycle metabolic regulation and tumor microenvironment. In normal cells, metabolic pathways are dynamically coordinated with cell cycle phases to supply energy and biosynthetic precursors: glycolysis, PPP, and glutamine metabolism support the G1/S transition; nucleotide and lipid synthesis peaks during S/G2; and AMPK-mediated catabolism fuels mitosis. In tumors, this coordination is disrupted by metabolic reprogramming, such as the Warburg effect, mutant IDH-driven 2-hydroxyglutarate accumulation, and mTOR hyperactivation, which not only accelerates proliferation but also reshapes the tumor microenvironment through lactate secretion, nutrient depletion, and immunosuppressive signaling, thereby promoting immune evasion and disease progression. Abbreviations: PPP, pentose phosphate pathway; PKM2, pyruvate kinase M2; PFK1, phosphofructokinase-1; GLS1, glutaminase-1; LDHA, lactate dehydrogenase A; SCF, Skp1-Cullin1-F-box ubiquitin ligase complex; AMPK, AMP-activated protein kinase; PFKFB3, 6-phosphofructo-2-kinase/fructose-2,6-biphosphatase 3; IDH, isocitrate dehydrogenase; ACLY, ATP citrate lyase; 2-HG, 2-hydroxyglutarate; APC/C, anaphase-promoting complex; CDH1, cadherin 1; F-6-P, D-fructose 6-phosphate; F-1,6-BP, fructose-1,6-bisphosphate; TTK, thymidine thymidine kinase; BUB1B, BUB1 mitotic checkpoint serine/threonine kinase B. (figure was created with Biorender.com)

Cell cycle progression is not only regulated by endogenous signals but also closely associated with the tumor microenvironment (TME) and metabolic reprogramming. The precise regulation and periodic fluctuation of metabolites such as nucleotides, amino acids, and lipids during the G1, S, and G2/M phases provide the necessary material foundation for processes like DNA replication and cell division [222]. This metabolic pacemaker is synergistically driven by Cyclin-CDK complexes and E3 ubiquitin ligases [223]. Its disruption in tumors can enhance abnormal proliferation, immune evasion, and therapeutic resistance (Fig. 6).

#### Cell cycle-coupled metabolic remodeling

The dynamic nature of metabolic regulatory networks is activated across different phases of the normal cell cycle. In early G1, Cyclin D3-CDK6 phosphorylates and inactivates pyruvate kinase M2 isoform (PKM2) and phosphofructokinase-1 (PFK1), thereby suppressing glycolysis [224]. Concomitantly, 6-phosphofructo-2-kinase/fructose-2,6-biphosphatase 3 (PFKFB3), glutaminase-1 (GLS1), and isocitrate dehydrogenase 3 redirect carbon flux toward the oxidative pentose phosphate pathway (PPP) [225]. This generates NADPH for redox homeostasis and ribose-5-phosphate for RNA synthesis [226]. In the late G1 stage, phosphorylated PKM2 forms low-activity dimers and partially translocates to the nucleus, where it functions as a protein kinase or transcriptional coactivator. PKM2 activation enhances the activity of β-catenin, *c-MYC*, and Hypoxia-inducible factor 1-alpha (HIF-1α) through these nuclear functions. This upregulates glucose transporters, PFK1, lactate dehydrogenase (LDH), and GLS1, promoting glycolysis and glutaminolysis to supply energy and biosynthetic precursors for cell growth [227].

During the onset of S phase, SCF targets PFKFB3 for degradation, which inhibits glycolysis and reroutes glucose flux to support pentose sugars and one-carbon units in nucleotide biosynthesis [228]. GLS1 levels are maintained by the sustained *c-MYC* activation to ensure nitrogen supply, while Cyclin E-CDK2 suppresses isocitrate dehydrogenase 1/2 (IDH1/2) activity, reducing α-ketoglutarate (α-KG) production and facilitating citrate export for cytoplasmic acetyl-CoA generation and lipid synthesis [229].

In the G2 phase, Cyclin A-CDK2 inhibits IDH1/2 and PFK1 while activating ACC, synergizing with ATP citrate lyase (ACLY) to drive de novo lipogenesis [227]. Malic enzyme 1 cooperates with the PPP to restore NADPH, thereby facilitating reductive biosynthesis [230]. During the late G2 phase, AMP-activated protein kinase (AMPK) detects energy levels and phosphorylates ACLY to preserve metabolic balance [231], whereas Cyclin B1-CDK1 upregulates mitochondrial oxidative phosphorylation (OXPHOS) genes, elevating ATP production to energize mitotic entry and the G2/M transition [232].

#### Metabolic dysregulation and malignant cell cycle progression

In tumor cells, aberrant coupling between metabolic reprogramming and cell cycle control sustains malignant proliferation [233]. The Warburg effect accelerates glycolysis through upregulation of glucose transporter 1 (GLUT1), hexokinase 2, and lactate dehydrogenase A (LDHA), thereby boosting lactate production [234]. Beyond ATP generation, lactate functions as a signaling metabolite, inducing histone H3K18 lactoylation and activating cell cycle genes such as thymidine kinase and BUB1B, thereby facilitating the G1/S transition and enhancing hypoxic adaptation in pancreatic cancer [235]. The PPP is amplified by elevated glucose-6-phosphate dehydrogenase under tumor protein p73, TAp73 isoform regulation, generating NADPH and ribose-5-phosphate, which are critical precursors for S-phase DNA synthesis [226]. GLS1-mediated glutaminolysis replenishes tricarboxylic acid cycle intermediates, supporting nucleotide biosynthesis and cell cycle advancement [236]. Citrate concentrations vary throughout the S/G2 phases; when converted to acetyl-CoA by ACLY, it supports lipid synthesis driven by FASN to meet the requirements of membrane biogenesis [237]. Disruptions from IDH mutations or citrate synthase inhibition impair this axis, halting cycle progression [238]. Moreover, the mTORC1 complex coordinates cellular processes [239]. Hyperactivated mTORC1 integrates metabolic signals to inhibit autophagy and boost the PPP [240]. This metabolic support allows cells to resist oxidative stress and fuel rapid proliferation, creating a powerful synergy between metabolic state and cell cycle progression.

#### Metabolism-TME-cell cycle interplay

Metabolic remodeling shapes the tumor microenvironment by coupling cell cycle abnormalities with immune suppression. Excess lactate accumulation acidifies the TME and impairs glycolysis and interferon-gamma secretion of CD8⁺ T cells [241–243]. It is also transported into regulatory T cells via monocarboxylate transporter 1 and converted to phosphoenolpyruvate [244]. This maintains TCR signaling, promotes PD-1 upregulation and nuclear factor of activated T cells-1 nuclear translocation, strengthens Treg immunosuppressive function, and is correlated with higher glycolytic demands in the late G1 phase [245]. By stabilizing HIF-1α and activating mTOR, lactate promotes M2 macrophage polarization in densely fibrotic pancreatic cancers. This results in the release of interleukin-10 and transforming growth factor-β, which inhibits effector T cell proliferation and traps them in G1 arrest [246].

Amino acid metabolism further contributes to immune evasion. Tryptophan catabolism by indoleamine 2,3-dioxygenase/tryptophan 2,3-dioxygenase generates indole derivatives that activate AHR signaling, promoting Treg differentiation while suppressing effector T cell activity [247]. AHR and nuclear factor erythroid 2-related factor 2 cooperate to preserve redox balance, enabling tumor cells to navigate the G2/M checkpoint [248]. To protect DNA replication and mitosis, this process depends on NADPH from the PPP during the S and G2 phases. Ferroptosis resistance is induced by dysregulated cell cycle control (such as p53 loss, AKT hyperactivation, and WEE1 perturbation), which increases solute carrier family 7 member 11 to lower lipid peroxidation [249]. Moreover, it initiates the release of DAMPs, recruits TAMs and myeloid-derived suppressor cells, and suppresses the activity of DC and NK cells, resulting in an immunosuppressive environment consistent with p53-deficient glycolytic reprogramming [250].

Autophagy represents another key node linking metabolism, immunity, and cell cycle control. Under cyclin-dependent stress, PI3K/AKT/mTOR elicits protective autophagy, with Vps34 orchestrating non-oxidative PPP flux to sustain Treg viability [251]. Conversely, autophagy blockade enhances CCL5-mediated NK cell recruitment, highlighting mTOR’s centrality in metabolic-immune-cell cycle integration [252]. Cancer-associated fibroblasts (CAFs) export lactate and glutamine to fuel tumor S-phase DNA synthesis, while tumor-derived Cyclin B1-CDK1 activation reciprocally stimulates CAF maturation and extracellular matrix deposition, amplifying fibrosis and metastasis [253]. This reciprocal loop converges with Cyclin B1-CDK1-driven OXPHOS reprogramming to collectively forge a pro-tumorigenic TME.

### Signaling pathways

**Fig. 7:**
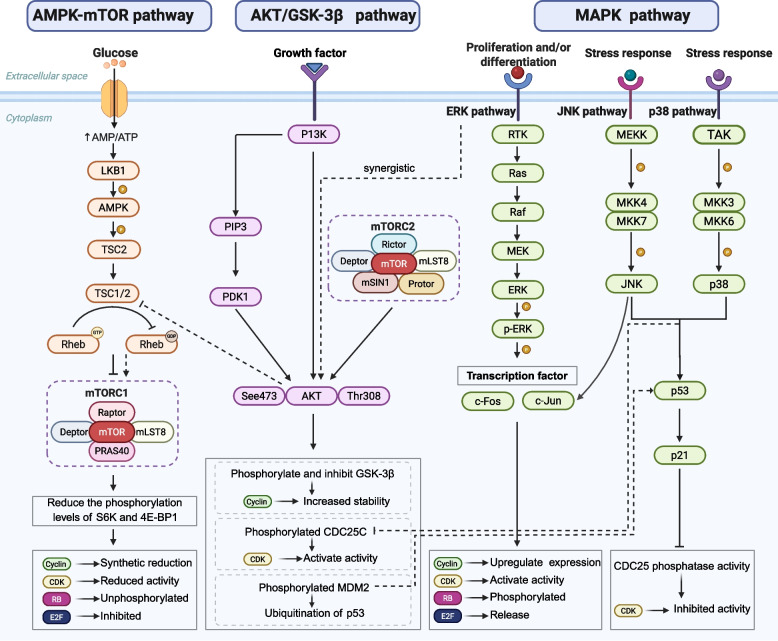
Regulatory mechanisms of core signaling pathways in the tumor cell cycle. AMPK, activated by LKB1 under energy stress, suppresses mTORC1 via TSC1/2, reducing S6K and 4E-BP1 phosphorylation to inhibit protein synthesis and induce cell cycle arrest. The AKT/GSK-3β pathway, activated by growth factors and mTORC2, promotes proliferation by stabilizing cyclins (via GSK-3β inhibition), enhancing anabolic metabolism, and triggering MDM2-mediated p53 degradation to bypass checkpoint arrest. Within the MAPK pathway, the ERK branch (Ras-Raf-MEK-ERK) drives G1/S progression through cyclin upregulation and Rb phosphorylation, whereas the stress-responsive JNK/p38 branch stabilizes p53, inducing p21 expression, and inhibits CDC25 to block CDK1/2 activation and enforce G1/S or G2/M arrest. Notably, these pathways engage in extensive crosstalk (dashed lines), forming an integrated signaling network that collectively dictates cell cycle fate in tumors. Abbreviations: LKB1, liver kinase B1; AMPK, AMP-activated protein kinase; TSC2, tuberous sclerosis complex 2; TSC1/TSC2, tuberous sclerosis complex 1/2; mTOR, mammalian target of rapamycin; RB, retinoblastoma protein; Akt, AKR mouse strain thymoma; GSK-3β, glycogen synthase kinase-3 beta; PI3K, phosphatidylinositol 3 kinase; RTK, receptor tyrosine kinase; RAS, rat sarcoma virus oncogene homolog; RAF, rapidly accelerated fibrosarcoma; MAPK, mitogen-activated protein kinase; ERK, extracellular signal-regulated kinase; MEK, MAPK/ERK kinase; MEKK, MAPK/ERK kinase kinase; MKK4, mitogen-activated protein kinase kinase 4; JNK, c-Jun N-terminal kinase; TAK, TGF-β-activated kinase; p38, p38 mitogen-activated protein kinase; CDC25, cell division cycle 25. (figure was created with Biorender.com)

The regulation of the cell cycle relies on the integration of multiple signaling pathways responding to nutrient availability, growth factors, energy status, and stress signals. In tumors, these pathways are frequently abnormally activated or inactivated, leading to cycle dysregulation and uncontrolled proliferation. The following discussion explains the impact of key signaling pathways on the regulation of cell cycle activity (Fig. 7).

#### AMPK-mTOR pathway: energy sensing and growth regulation

AMPK is a chief cellular energy sensor, activated by ATP depletion and an elevated AMP/ATP ratio to restore energy balance [254, 255]. Its activation is mediated by liver kinase B1, which promotes tuberous sclerosis complex 1/2 (TSC1/TSC2) complex formation by phosphorylating TSC2, which inhibits mammalian target of rapamycin (mTOR) activity [256]. mTOR acts as a central regulatory factor that integrates nutrition, energy, and growth factor signaling [257, 258]. AMPK-mediated mTOR suppression reduces phosphorylation of downstream effectors such as S6K and 4E-BP1, leading to decreased Cyclin D1 translation, reduced CDK4/6 activity, and G1-phase arrest [259]. Notably, bacterial cyclodipeptides (CDPs) trigger mitochondrial stress in cervical cancer HeLa cells, activating AMPK, reinforcing TSC1/TSC2 function, and thereby inhibiting mTOR pathway-mediated protein translation [260]. This action induces G1 phase arrest and triggers apoptosis. Collectively, these findings emphasize the impact of the participation of the AMPK-mTOR axis in tumorigenesis and highlight the possible function of this pathway for therapeutic interventions.

#### Mitogen-activated protein kinases (MAPK) pathway: multifaceted regulation of cell fate

The MAPK cascade serves as a multifaceted signaling hub that governs cell proliferation, differentiation, stress adaptation, and apoptosis, substantially impacting tumorigenesis [261–263]. It encompasses three core branches, such as extracellular signal-regulated kinase (ERK), c-Jun N-terminal kinase (JNK), and p38, each attuned to specific stimuli and eliciting distinct cellular outcomes [264–266]. The ERK pathway is typically activated by growth factors through the Ras-Raf-MEK-ERK cascade [267]. Activated ERK translocates to the nucleus, where it phosphorylates transcription factors such as c-Fos and c-Jun, upregulating Cyclin D1 expression [268]. This subsequently activates CDK4/6, facilitating the G1/S transition and encouraging cell proliferation [269]. Conversely, the JNK pathway exhibits a context-dependent duality, fostering oncogenesis in some environments while inducing apoptosis or cycle arrest in others [270]. By phosphorylating p53 and c-Jun, JNK safeguards genomic integrity; its suppression correlates with elevated mutational burden, including deletions and insertions [271]. JNK also modulates Rb and E2F activity, thereby shaping S-phase progression. The p38 pathway primarily detects inflammatory cytokines, oxidative stress, and DNA damage [272, 273], It frequently interacts with JNK to regulate the cell cycle. Upon DNA damage, both factors jointly promote p53 phosphorylation and stabilization, induce p21 expression, and inhibit CDC25 phosphatase activity, thereby blocking CDK1/2 activation and inducing G1/S or G2/M phase arrest [274]. This creates a time window for DNA repair, preventing the accumulation of mutations. Collectively, the MAPK network integrates proliferative and stress signals to preserve homeostasis and restrain malignant transformation.

#### AKT/GSK-3β Pathway: growth promotion and cycle drive

AKT is a central serine/threonine kinase within the PI3K-AKT signaling axis and regulates cell survival, metabolism, and cell cycle progression [275, 276]. The full activation of the cell cycle requires PI3K-dependent recruitment to the plasma membrane, followed by PDK1-mediated phosphorylation at Thr308 and mTORC2 at Ser473 [277]. When AKT is activated, the cell cycle progression is promoted and is driven through multiple linked pathways. First, it phosphorylates GSK-3β at Ser9, inactivating it and preventing proteasomal degradation of Cyclin D1. Stabilized Cyclin D1 assembles active CDK4/6 complexes, driving Rb hyperphosphorylation and E2F release to initiate S-phase entry [278]. Second, AKT phosphorylates MDM2, which increases its nuclear localization and promotes the ubiquitination and degradation of p53. This process inhibits the tumor-suppressing functions of p53, such as cell cycle arrest and apoptosis [279]. Third, AKT directly phosphorylates CDC25C, enhancing its phosphatase activity and suppressing the inhibition of CDK1, thereby facilitating mitotic entry even under stressful conditions [280]. Notably, the effects of AKT/GSK-3β signaling vary significantly depending on the specific cell type, cellular localization, and the type of stimulus received. For instance, the GSK-3β inhibitor lithium chloride promotes nephroprotection in diabetic nephropathy by stabilizing β-catenin but induces G2 arrest in HK-2 cells via the same axis, illustrating its bimodal, tissue-dependent regulation [281].

#### Crosstalk and dynamic regulation of cell cycle signaling

It should be highlighted that cell proliferation is governed by a dynamic, interconnected network of signaling pathways. Under energy stress, AMPK phosphorylates TSC2, which inhibits mTOR and limits the synthesis of Cyclin D1, reducing CDK4/6 activity and stimulating G1 arrest [259]. In contrast, during normal growth, ERK and AKT cooperate to drive G1 progression: ERK activates transcription factors to enhance Cyclin D1 gene expression, while AKT stabilizes Cyclin D1 protein by inhibiting GSK-3β, working together to maintain its nuclear accumulation and driving cells through the G1/S checkpoint [1]. Following DNA damage, MAPK family members like p38 and JNK are activated and work to arrest the G1/S or G2/M phases by phosphorylating p53 and inhibiting CDC25 phosphatases [282]. AKT antagonizes these arrest signals by phosphorylating CDC25C or promoting p53 degradation, thereby sustaining progression [282]. Furthermore, growth factor-activated AKT and ERK synergistically enhance mTOR signaling, further promoting cell cycle progression [283].

Together, the balance and interplay among the AMPK-mTOR, MAPK, and AKT/GSK-3β pathways determine cell cycle outcomes in response to metabolic status, growth cues, and stress signals. Disruption of this equilibrium is a hallmark of tumorigenesis and provides a conceptual framework for therapeutic strategies aimed at restoring cell cycle control. Furthermore, emerging pathways such as Wnt/β-catenin and Hippo have also been implicated in cycle regulation, although this paper does not delve into those aspects.

## Dysregulation of the cell cycle in cancer: from mechanism to biomarker

Cancer development is marked by the breakdown of the normal regulation of the cell cycle, manifested by the hyperactivation of positive regulators, the inactivation of negative regulators, and the failure of the cell cycle checkpoints. These alterations collectively promote genomic instability and uncontrolled proliferation [284–286]. Elucidating these mechanisms not only clarifies the pathological basis of tumorigenesis but also facilitates the identification of diagnostic and prognostic biomarkers and therapeutic targets (Table 1).

**Table 1:** Mechanisms of cell cycle dysregulation and their application as biomarkers in cancer

Dysregulation mechanism category	Key molecules/pathways	Dysregulation mechanisms of common cancer types	Common cancer types	Clinical significance as biomarkers	References
Hyperactivation of positive regulators	Cyclin D1	Gene amplification, overexpression	Anaplastic lymphoma; High-grade endometrial stromal sarcoma; Bladder cancer; Melanoma;	Diagnostic marker (Cyclin D1 has been established as a primary diagnostic marker for diseases such as mantle cell lymphoma and high-grade endometrial stromal sarcoma); Poor prognostic marker (high Cyclin D1 expression may indicate high proliferative activity and poor prognosis)	[287]
	CDK4/6	Overexpression	Ovarian cancer; Gastric cancer; Lung adenocarcinoma; Glioblastoma; Melanoma	Diagnostic marker (high expression of CDK4/6 in cancerous areas); poor prognostic marker (excessive CDK4/6 levels significantly shorten overall survival)	[288]
	CDK3	Gene amplification	Hepatocellular carcinoma; Colorectal cancer; Nasopharyngeal carcinoma	Diagnostic marker (CDK3 overexpressed in cancerous areas), CDK3 positively correlates with cervical lymph node metastasis and advanced T staging	[289, 290]
	CDK7	Overexpression	Osteosarcoma; Non-small cell lung cancer; Esophageal squamous cell carcinoma; Head and neck squamous cell carcinoma	Diagnostic marker (high CDK7 expression in cancerous areas), poor prognostic marker (patients with high CDK7 expression exhibit poorer overall survival and disease-free survival and show a positive correlation with metastasis/recurrence)	[291, 292]
	Cyclin E-CDK2	Overexpression	Breast cancer; Renal cell carcinoma	Diagnostic marker (Cyclin E-CDK2 is highly co-expressed in cancerous areas), poor prognostic marker Cyclin E-CDK2 is highly co-expressed, indicating active tumor proliferation and poor prognosis	[293]
Inactivation of tumor suppressors	p16	Downregulation/absence, abnormally high expression	Cervical cancer; Head and neck squamous cell carcinoma; Glioma; Pancreatic cancer; Melanoma	p16 protein deficiency may serve as a prognostic/subtyping indicator for melanoma, pancreatic cancer, etc. Abnormally high p16 expression in head and neck/cervical cancer can be utilized for diagnostic subtyping	[294, 295]
	p21	Nuclear expression retention	Colorectal cancer	p21 nuclear expression retention suggests the p53 pathway may remain intact, serving as a positive surrogate marker for chemotherapy sensitivity	[296]
	p27	Low expression	Breast cancer; Prostate cancer	p27 nuclear expression reduction with cytoplasmic mislocalization indicates poor differentiation and increased risk of endocrine resistance	[297, 298]
Checkpoint failure and genomic instability	p53	*TP53* gene mutation, p53 downregulation, inactivation	High-grade serous ovarian cancer; Head and neck squamous cell carcinoma; Metastatic colorectal cancer; Triple-negative breast cancer	Predicting DNA damage-related relative sensitivity to chemotherapy/radiotherapy as an independent adverse prognostic indicator	[299, 300]
	Telomere length	Telomere dysfunction	Breast Cancer; Colorectal Adenoma	Short telomere length indicates an increased risk of aggressive recurrence within 5 years and an elevated risk of high-grade intraepithelial neoplasia	[301, 302]
	TRF2	Low expression	Breast Cancer; Colorectal Adenoma	Low TRF2 expression correlates with high-grade tumors, Ki-67 > 30%, positive lymph node metastasis, and poor prognosis in microsatellite stable patients	[303]

### Hyperactivation of positive regulators

Cell cycle progression is driven by cyclins and CDKs, which assemble into Cyclin-CDK complexes activated by CAK. However, the multilayered and synergistic breakdown and dysregulation of the cell cycle in tumors forms the cornerstone for the pathogenesis. This process has significant biological implications and is widely useful for diagnosis, classification, and prognostic assessment [304].

Cyclin D1 serves as a crucial regulatory molecule that initiates the G1 phase and is frequently overexpressed in diverse malignancies [305]. In mantle cell lymphoma, the t(11; 14) (q13; q32) translocation drives constitutive Cyclin D1 overexpression, with a Cyclin D1/GAPDH ratio ≥ 100 via real-time RT-PCR established as the diagnostic positivity threshold [287]. In gynecologic tumors, strong nuclear Cyclin D1 expression is characteristic of YWHAE-FAM22 fusion and positive high-grade endometrial stromal sarcoma, distinguishing it from low-grade endometrial stromal sarcoma, leiomyosarcoma, and carcinosarcoma [287, 306]. Furthermore, Cyclin D1 overexpression is also observed in approximately 51.6% of bladder urothelial carcinomas [307]. In melanoma, Cyclin D1 levels are not only markedly upregulated but also remain elevated with greater tumor invasion depth, highlighting its effectiveness as a tool for differential diagnosis and a prognostic tool [308]. Overall, the elevated expression of Cyclin D1 usually indicates rapid growth and a poor prognosis.

In addition to Cyclin D1, overexpression of CDK4/6 is common in solid tumors. Analyses of 20 prevalent solid tumor types reveal significantly elevated CDK4/6 mRNA levels in 14 of them compared to matched normal tissues [288]. Notably, in breast cancer, elevated CDK4/6 expression correlates with reduced overall survival [309]. Particularly, over 70% of ovarian cancer cases show nuclear CDK4/6 positivity, whereas normal fallopian tube or ovarian epithelium shows very little expression, demonstrating its remarkable tumor specificity [310]. Emerging evidence also highlights the oncogenic role of CDK3. Gene amplification or aberrant expression of CDK3 accelerates G1/S phase transition and induces centrosome overduplication, thereby promoting genomic instability. In colorectal cancer, CDK3 mRNA is markedly upregulated [289]. In nasopharyngeal carcinoma, CDK3 positivity stands at 67%, with expression positively associated with cervical lymph node metastasis and advanced T stage, positioning it as a promising prognostic marker [290]. Moreover, as a key member of the CDK-CAK complex, CDK7 plays dual roles in transcriptional regulation and CDK activation, and its upregulation is observed in a variety of cancers. For example, high nuclear CDK7 expression is detected in 95.6% of osteosarcomas and correlates with poor prognosis, metastasis, and recurrence [292]. Similar associations between elevated CDK7 levels and adverse outcomes have been reported in non-small cell lung cancer, esophageal squamous cell carcinoma, and head and neck squamous cell carcinoma, particularly in patients with lymph node metastasis [311–313].

Dysregulation of the Cyclin E-CDK2 complex represents another major mechanism of aberrant cell cycle activation. Inactivation of the E3 ubiquitin ligase F-box and WD repeat domain containing 7 enhances Cyclin E stability, resulting in its intracellular accumulation, which induces replication stress and genomic instability [293, 314]. This pathway frequently works together with oncogenic signaling pathways, including the *Ras* and *ErbB2* genes, thus promoting tumor progression and possibly enhancing the self-renewal ability of cancer stem cells [315]. Both Cyclin E and CDK2 are markedly elevated in colorectal cancer [316].

### Inactivation of tumor suppressors

Negative regulators of the cell cycle primarily rely on the Rb proteins and *CKIs* [317]. *CKIs* primarily consist of two major families, which include *INK4* and *CIP/KIP* [318, 319]. The *INK4* family (Inhibitor of *CDK4*), including *p16INK4a*, *p15INK4b*, *p18INK4c*, and *p19INK4d* (also termed *p16*, *p15*, *p18*, and *p19*) is encoded by the *CDKN2A*, *CDKN2B*, *CDKN2C*, and *CDKN2D* genes, respectively [320]. The *CIP/KIP* family includes *p21CIP1/WAF1*, *p27KIP1*, and *p57KIP2* (also known as *p21*, *p27*, and *p57*) and is encoded by the *CDKN1A*, *CDKN1B*, and *CDKN1C* genes, respectively [321]. These proteins function as tumor suppressors, and their loss or functional impairment is a common initiating event in tumorigenesis.

Tumor suppressor inactivation occurs through diverse mechanisms, including gene mutations, allelic loss, promoter hypermethylation, and aberrant protein degradation. For example, *CDKN2A* gene loss is common in gliomas [322], pancreatic cancers [323], and melanomas [324], resulting in the absence of its encoded p16 protein. This suppresses the inhibition of the Cyclin D-CDK4/6 complex, resulting in the continued phosphorylation of the Rb protein. Consequently, cells evade the G1/S checkpoint, accelerating malignant transformation [294]. Conversely, p16 overexpression is characteristic of HPV-associated cervical cancer and head and neck squamous cell carcinoma, where it serves as a surrogate biomarker for viral infection and has diagnostic and prognostic value [295]. Members of the CIP/KIP family also show cancer-specific alterations. In colorectal cancer, preserved nuclear p21CIP1/WAF1 expression reflects an intact p53 pathway and predicts sensitivity to chemotherapy [296]. In breast cancer and prostate cancer, p27KIP1 frequently exhibits reduced nuclear expression and cytoplasmic translocation, which suppresses its inhibitory effect on Cyclin A/E-CDK2. This is associated with poor differentiation, resistance to endocrine therapy, and an unfavorable prognosis [297, 298].

Collectively, the convergence of oncogenic activation and tumor suppressor inactivation profoundly disrupts cell cycle homeostasis, driving malignant transformation and progression. Restoration or functional mimicry of tumor suppressor pathways, therefore, remains a central objective of targeted cancer therapy.

### Checkpoint failure and genomic instability

Cell cycle checkpoints ensure genomic integrity by monitoring DNA damage and chromosome segregation. Their dysfunction induces CIN and microsatellite instability, fostering tumor heterogeneity and disease progression [325, 326].

Mutations in *TP53* represent the most prevalent cause of checkpoint dysfunction in human tumors. As a guardian of the genome, *TP53* induces cell cycle arrest, DNA repair, or apoptosis through transcriptional activation of genes such as *CDKN1A*, following genotoxic stress [327]. Research indicates that inactivating *TP53* mutations occur in more than 50% of human tumors, allowing cells to proliferate despite accumulating DNA damage [328]. In high-grade serous ovarian cancer [329], head and neck squamous cell carcinoma [330], metastatic colorectal cancer [331], and triple-negative breast cancer [332], p53 loss is commonly associated with G1/S and G2/M checkpoint dysfunction and a high CIN phenotype. Notably, p53 loss also serves as a predictive biomarker, indicating tumor sensitivity to DNA-damaging therapies such as platinum-based chemotherapy or radiotherapy.

Telomere dysfunction constitutes another major driver of CIN. Telomere attrition or reduced telomeric repeat-binding factor 2 (TRF2) expression triggers persistent DNA damage signaling, circumvents senescence, and promotes end-to-end chromosomal fusions and structural rearrangements [333]. Clinically, shortened leukocyte telomere length coupled with low TRF2 expression in breast cancer is associated with aggressive recurrence, high histologic grade, elevated Ki-67, and lymph node metastasis [301, 302]. In colorectal cancer, a ≥ 50% reduction in TRF2 levels triples breakage-fusion-bridge cycles, predominantly in microsatellite-stable tumors with poor prognosis [303]. Moreover, colorectal adenomas with telomere lengths under 1.2 kb face a higher risk of advancing to high-grade intraepithelial neoplasia [334]. Centrosome abnormalities further contribute to CIN [335]. For instance, *PLK4* haploinsufficiency drives centrosome amplification and multipolar mitoses, an effect aggravated in cells lacking the p53 protein. This occurs because p53 normally functions as a tumor suppressor by triggering cell cycle arrest or apoptosis in cells with supernumerary centrosomes, a mechanism that is bypassed in p53-null cells [336]. Replication stress simultaneously induces telomere dysfunction and centrosome amplification, which then form a pathological feedback loop, driving a profound level of genetic instability. Furthermore, epigenetic dysregulation also governs CIN [337]. Promoter hypermethylation of mismatch repair genes, such as *mutl homolog 1*, induces MSI in 10–15% of colorectal cancers [338]. From a clinical perspective, MSI status and homologous recombination deficiency scores are established predictors of response to immune checkpoint blockade and poly (ADP-ribose) polymerase (PARP) inhibitors [339]. Furthermore, CIN quantification, via copy number variation or arm-level alteration metrics, is gaining traction as a prognostic and therapy-guiding biomarker in clinical trials [340].

In summary, checkpoint failure, telomere erosion, centrosome aberrations, and epigenetic silencing rarely act alone. Rather, they interact synergistically throughout tumor evolution, collectively sculpting the CIN phenotype, dictating tumor behavior, and modulating treatment response. Future investigations should analyze the drivers of complex interactions between these pathways involved in the cell cycle to advance precision oncology.

## Therapeutic intervention strategies targeting cell cycle signaling pathways

Recent advances in understanding cell cycle regulation, coupled with innovations in drug development, have driven significant progress in targeted therapies that disrupt cell cycle signaling. Current strategies primarily encompass three key directions: direct inhibition of CDKs, synthetic lethality, and combination therapy regimens (Table 2). These approaches effectively induce tumor cell cycle arrest and apoptosis while significantly increasing specificity and the potential to overcome drug resistance. Notably, therapies involving naturally derived products are playing an increasingly pivotal role in treatment strategies, owing to their structural diversity and multi-target regulatory capabilities. Many naturally derived compounds serve not only as novel CDK inhibitors or synthetic lethality inducers but also as synergistic enhancers that amplify the antitumor efficacy of existing treatments, all while demonstrating reduced toxicity and fewer side effects. The systematic identification and mechanistic understanding of these substances have the potential to substantially advance precision oncology.

**Table 2:** Clinical evidence summary of three major therapeutic strategies targeting cell cycle regulatory networks

Joint strategy category	Representative drugs/natural products	The main mechanism	Research model/clinical stage	Target cancer species	Identifier	Injection mode	Key efficacy data/effects	Main challenges	Reference
CDK4/6 inhibitors	Palbociclib	Block G1/S or G2/M conversion	Have undergone a complete clinical trial	Breast cancer	NCT02107703	Oral	Median PFS was 24.8 months; ORR was 55.3%	Drug resistance	[341]
CDK2/5/9 inhibitor	Fadraciclib	Inhibition of CDK2 leads to a decrease in E2F1 transcription factors, induces G1 phase arrest, inhibits CDK9, and prevents phosphorylation at the Ser2 site of the RNAPII C-terminal domain, thereby blocking transcriptional extension and inducing apoptosis in the AML/ALL preclinical model	Phase I/II trials are underway	Leukemia	CYC065	Oral	TGI was 97–99%	Continuous administration is required to maintain a balance between MCL-1 inhibition and patient tolerance	[342–344]
CDK7 inhibitor	Samuraciclib	By reversibly occupying ATP binding sites, anti-proliferative effects can be achieved, and acquired resistance to HER2 inhibitors can be reversed	The 3D sphere models of TNBC, SCLC, and AML showed significant anti-proliferative effects and were in the phase I/II clinical research stage	Triple-negative breast cancer; Small cell lung cancer	CT7001	Oral	The median PFS was 13.8 months	Gastrointestinal toxicity	[345]
Endogenous molecule circ RNA	Circ_0007379	It can inhibit CDK2 activity or block Cyclin-CDK assembly by assisting miRNA to form a ternary complex with CDK2/p21, transcription/translation, and other multiple mechanisms, thereby blocking cell cycle arrest in the G1/S or G2/M phase	Current clinical studies mainly focus on vaccines or rare diseases. Its application in the field of cancer is still in the preclinical stage	Colorectal cancer	_	In vitro cell transfection, in vivo intratumoral injection, or systemic administration	TGI was 65–75%	It is difficult to enter the clinical trial stage	[346]
Natural product	Saikosaponin D	By up-regulating p21/p27 and down-regulating Cyclin D1-CDK4/6 or Cyclin B1-CDK1 activity, tumor cells are arrested in the G0/G1 or G2/M phase, and the EGFR/p38 signaling pathway is inhibited to inhibit proliferation and induce apoptosis	In vitro and animal experiments have confirmed its anti-tumor activity in a variety of tumor models, but so far, saikosaponin D has not entered any registered phase I clinical trial	Breast cancer	_	Intraperitoneal injection or oral administration	Inhibition of TNF-α-activated monocyte adhesion to HUVEC, IC50 was 1.8–3.0 μM	Insufficient data on efficacy and safety when used alone	[347, 348]
RSK2 inhibitor	SL0101	By inhibiting the downstream ERK signaling pathway to regulate cell proliferation and metastasis, it has shown efficacy in breast cancer and melanoma xenograft models	Has not yet entered clinical trials	Breast cancer and melanoma	_	Intraperitoneal injection or oral	Significantly inhibit breast cancer cell proliferation/survival/migration inhibition	In vivo pharmacokinetic defects	[349]
Chinese medicine	Coix seed	It exerts anti-tumor and attenuated synergistic effects through multi-pathway synergy (possibly including down-regulation of CDK4/6 expression)	Most of them are small sample studies or preclinical experiments	Non-small cell lung cancer; colon cancer	_	Intravenous injection	Inhibition of tumor cell survival rate	It has been approved, but the effective concentration is high and the target specificity is high	[350]
Chinese medicine	Silymarin	Through anti-oxidative stress and induction of G1 phase arrest	At present, there is no experimental evidence for its direct inhibition of CDK activity	Liver cancer	_	Oral	Reduce mortality, bilirubin, GGT, and AST/ALT levels	Low bioavailability	[351, 352]
PARP inhibition + HRR defect	Olapaly	PARP inhibition leads to SSB accumulation, replication fork collapse, irreparable DSB, and cell death	Has been fully into the clinical	Breast Cancer	NCT03604783	Oral/intravenous injection	ORR was 17%, and PFS was 9.3 months	Standard treatment has been approved, but drug resistance is common	[353]
WEE1 inhibitor + ATRX mutation/p53 defect	Adavosertib	Destruction of the G2/M checkpoint can force cells carrying DNA damage to enter mitosis in advance, thereby inducing a mitotic disaster	Further clinical development has been suspended, and the focus of research has shifted to second-generation WEE1 inhibitors	Colorectal cancer	AZD1775	Oral	The median PFS was 13.8 months	Drug resistance and efficacy-toxicity ratio are not ideal	[354]
ATR inhibitors + p53 inactivation/ATM defects	Berzosertib	ATR inhibition blocks ATR-Chk1 signaling, CDC25C is activated, CDK1/2 is prematurely activated, and cells are forced into mitosis when DNA damage is not repaired	Clinical Phase I/II Trials	Small cell lung cancer	VX-970	Oral or intravenous infusion	The objective remission rate of chemotherapy can reach 30–50%	Poor efficacy	[355–357]
Natural products + HRR defects	Paclitaxel, cisplatin	ROS-mediated oxidative stress can destroy G2/M checkpoint integrity, inhibit homologous recombination repair, and amplify DNA damage signals	The pre-study is still in the stage of clinical verification, and there is no phase III evidence	Esophageal squamous cell carcinoma	-	Intravenous infusion	The radiosensitivity of the tumor was improved	ROS-related toxicity	[358]
PI3K/AKT/mTOR pathway activation + CDK4/6 inhibitor	Alpelisib/Gedatolisib + Palbociclib	The activation of the PI3K/AKT/mTOR pathway can upregulate Cyclin D and weaken the efficacy of CDK4/6 inhibitors	There is still a lack of phase III evidence	Breast cancer	NCT05501886	Oral	Increased PFS	Toxicity superposition and drug resistance mechanisms are diverse	[359]
Anti-EGFR + CDK4/6 inhibitors	Cetuximab + Reboxetine	In the preclinical model, synergistic anti-tumor effects were achieved by simultaneously blocking the EGFR-MAPK and PI3K pathways	In the preclinical model	Breast cancer; colorectal cancer	_	Injection + Oral	Ki-67 decreased to below 30% of baseline	Toxicity superposition	[360]
CDK4/6 + anti-PD-1/BET inhibitors	Ribociclib + Pembrolizumab + JQ1	CDK4/6 inhibitors can inhibit DNMT1 to reverse T cell exhaustion, induce immune memory in combination with anti-PD-1, and upregulate PD-L1 in combination with BET inhibitors, thereby enhancing ICI sensitivity	Only seen in preclinical studies	TNBC	_	Injection + Oral	Tumor regression	The balance between toxicity and efficacy is difficult to solve	[361]
CDK2, CDK4, and CDK6 inhibitors	PF-06873600	As a three-target inhibitor, a CDK2 inhibitor simultaneously inhibits CDK2, CDK4, and CDK6, which is expected to overcome the resistance of ER + advanced breast cancer to AI and CDK4/6i	It has entered phase I/II clinical practice, and the development has been suspended due to large toxicity problems	Breast cancer	PF-06873600	Oral	The Ki-67, pRb score, and ctDNA level of tumor tissue were decreased	Toxicity is a big problem	[362]
DDR + CDK4/6 inhibitors	Gemcitabine + Palbociclib	Paboxetine can induce G1 phase arrest to synchronize tumor cells into S phase, which maximizes the killing efficiency of gemcitabine	It has entered the phase I/II clinical trial stage	Ovarian cancer	NCT02264678	Injection + Oral	PFS was 27.7 weeks	Toxicity limitation	[363, 364]
Natural products	Ginsenoside Rg3	Adjuvant therapy by targeting the tumor microenvironment and cancer cells	Phase II-III clinical trials	Non-small cell lung cancer; hepatocellular carcinoma	NCT02724358	Oral	The clinical efficacy was significantly improved, and the OS was significantly prolonged by about 15%	It has not been approved as a single drug	[365]
Natural products + kinase inhibitors	Curcumin + Sorafenib	The combination can regulate metabolism and tumor microenvironment and enhance the anti-tumor effect	Only the phase I safety study was completed, and the phase II/III confirmatory test was not entered	Liver cancer	_	Oral	Prolong OS	Curcumin inhibits CYP3A4 metabolic enzymes, which may increase the blood concentration of sorafenib and aggravate the toxicity	[366, 367]
Natural products + CDK inhibitors	Resveratrol + Quercetin	It can activate the p53 pathway and overcome the resistance caused by Rb deletion	They have not yet entered clinical trials	Colon cancer	_	Oral	The objective remission rate was 65%	Very low bioavailability	[368]
Isothiocyanate compounds, PARP inhibitors	Sulforaphane + Olaparib	The combination can enhance the synthetic lethal effect by enhancing DNA damage	Did not enter the clinical trial stage	Ovarian cancer	_	Oral	Increased DNA damage marker H2AX foci	Very low bioavailability	[369]

*Abbreviations*: *PFS* progression-free survival, *ORR* objective response rate, *TGI* tumor growth inhibition rate, *IC50* half maximal inhibitory concentration, *GGT* gamma-glutamyl transferase, *AST/ALT* aspartate aminotransferase/alanine aminotransferase, *OS* overall surviva

### Direct cell cycle kinase inhibitors

Targeting cell cycle kinases, particularly CDKs, represents a cornerstone of precision oncology. Selective inhibition of CDK4/6, CDK2, CDK7, and CDK9 can block G1/S or G2/M transitions, thereby suppressing tumor proliferation. Currently, four selective CDK4/6 inhibitors, palbociclib (approved by the FDA in 2015), ribociclib (approved by the FDA in 2017), abemaciclib (approved by the FDA in 2017), and China’s domestically developed dalpiciclib (approved by the NMPA in 2021), have been approved for HR +/HER2- advanced breast cancer [341]. In recent years, several next-generation CDK inhibitors have been under clinical evaluation. Fadraciclib (CYC065), a multi-target inhibitor of CDK2/5/9, induces apoptosis in preclinical models of acute myeloid leukemia (AML) and acute lymphoblastic leukemia (ALL) and is currently in phase I/II trials [342]. Samuraciclib, a selective CDK7 inhibitor, reversibly occupies the ATP-binding site, demonstrating significant antiproliferative effects in 3D spheroid models of triple-negative breast cancer (TNBC), small-cell lung cancer (SCLC), and AML. It can also reverse acquired resistance to HER2 inhibitors and is currently in phase I/II clinical trials [345].

Additionally, endogenous molecules and natural products also demonstrate unique potential in modulating CDK activity. Circular RNA can inhibit CDK2 activity or disrupt Cyclin-CDK assembly by regulating transcription, translation, or miRNA-mediated interactions, leading to G1/S or G2/M arrest [346]. For example, downregulation of circ_0007379 correlates with poor prognosis and advanced stage in colorectal cancer, suggesting its potential as an RNA-based therapeutic target [370]. Despite their promise, circRNA therapeutics for cancer remain at a preclinical stage, with current clinical studies mainly focused on vaccines or rare diseases [371].

The natural product saikosaponin D induces G0/G1 or G2/M arrest by upregulating p21/p27 and downregulating Cyclin D1-CDK4/6 or Cyclin B1-CDK1 activity while also inhibiting epidermal growth factor receptor (EGFR)/p38 signaling pathway [347]. Although its antitumor efficacy has been demonstrated in vitro and in animal models, clinical translation is limited by the lack of human data [372]. Similarly, the Ribosomal S6 kinase 2 (RSK2) inhibitor SL0101 (derived from Kaempferia parviflora) suppresses the ERK signaling pathway [349] and inhibits proliferation and metastasis in breast cancer and melanoma xenograft models but has not yet entered clinical trials.

In addition, some approved Chinese herbal medicine injections indirectly influence cell cycle regulation. Coix seed extract injection has shown clinical benefit as an adjuvant therapy in non-small cell lung cancer and colorectal cancer, potentially through downregulation of CDK4/6 expression [350]. Silymarin has also demonstrated adjuvant potential in hepatocellular carcinoma by inducing G1 arrest and alleviating oxidative stress, although direct CDK inhibition has not been conclusively demonstrated [351, 352]. Overall, the multi-target characteristics of natural products help mitigate the risks of resistance. Future research should focus on improving bioavailability through nanoformulations or structural modifications to accelerate clinical translation.

### Exploiting synthetic lethality and checkpoint vulnerabilities

Synthetic lethality exploits tumor-specific genetic defects (such as abnormalities in DNA damage response pathways) to effectively eliminate cancerous cells while leaving normal cells unharmed [373]. Poly (ADP-ribose) polymerase inhibitors (such as PARPIs and olaparib) exemplify this approach [374]. In tumors with homologous recombination repair (HRR) defects caused by BRCA1/2 mutations, PARP inhibition prevents single-strand breaks from being repaired. These breaks then convert into lethal double-strand breaks during replication, triggering replication fork collapse [353]. Consequently, PARPIs have received clinical approval for multiple tumor types.

Beyond PARP-based strategies, the G2/M checkpoint represents a critical vulnerability in tumor cells experiencing replicative stress. WEE1 kinase, as a key negative regulator of this checkpoint, and its inhibitors, such as adavosertib, can trigger cells carrying damaged DNA to prematurely enter mitosis, thereby inducing mitotic catastrophe [375]. Studies demonstrate that WEE1 inhibitors exhibit significant synergistic lethal effects in ATRX-mutant gliomas or p53-deficient solid tumors [354]. Furthermore, the ATR-CHK1-WEE1 signaling pathway, which plays a fundamental role in the replication stress response, is significantly activated in tumors lacking p53, acting as an essential compensatory mechanism to ensure cell survival [202]. Consequently, ATR inhibitors (such as berzosertib) also form a synthetic lethal relationship with p53 inactivation or ATM defects. Clinical phase I/II trials indicate that ATR inhibitors achieve objective response rates of 30–50% in small cell lung cancer, either as monotherapy or in combination with chemotherapy [355–357].

Natural products can further potentiate synthetic lethality. For instance, the combination of paclitaxel and cisplatin disrupts G2/M checkpoint integrity through ROS-mediated oxidative stress, inhibits homologous recombination repair, and amplifies DNA damage signals. This combination works in conjunction with chemoradiotherapy for esophageal squamous cell carcinoma, greatly improving the tumor’s sensitivity to radiation [358].

Although synthetic lethality theoretically offers the benefit of reduced toxicity to normal tissues, its application in clinical settings encounters numerous obstacles. These include instability in synthetic lethality pairing due to tumor genetic heterogeneity, toxicity arising from off-target inhibition of normal cellular compensatory pathways, and acquired resistance mechanisms such as HRR functional restoration or alternative checkpoint activation.

### Combination therapies for enhanced efficacy and overcoming resistance

While single-target therapies that target the cell cycle have made progress in cancer treatment, their efficacy is often limited by tumor heterogeneity and the activation of compensatory pathways, which can lead to drug resistance. For instance, CDK4/6 inhibitors substantially enhance the prognosis of HR +/HER2- breast cancer, but resistance problems are prevalent [376]. Consequently, rational combination strategies that exploit pathway crosstalk and synthetic vulnerabilities are increasingly pursued to enhance efficacy, reduce dose-limiting toxicity, and delay resistance emergence.

In HR +/HER2- advanced breast cancer, hyperactivation of the PI3K/AKT/mTOR pathway promotes Cyclin D expression and attenuates sensitivity to CDK4/6 inhibition [377]. Alpelisib (targeting PIK3CA mutations, SOLAR-1 study) or gedatolisib (in early-stage clinical trials) combined with CDK4/6 inhibitors demonstrated synergistic potential in exploratory studies, though phase III evidence remains lacking [359]. Combining cetuximab with CDK4/6 inhibitors such as palbociclib shows synergistic antitumor effects in preclinical models by simultaneously blocking the EGFR-MAPK and PI3K pathways [360]. Regarding immune microenvironment modulation, CDK4/6 inhibitors suppress DNMT1 to reverse T-cell exhaustion. In TNBC mouse models, they induce immune memory when combined with anti-PD-1 and upregulate PD-L1 when paired with BET inhibitors, mechanistically enhancing ICI sensitivity [361]. To address compensatory CDK2 activation following CDK4/6 inhibitor resistance, multi-pathway blockade strategies combining CDK2 inhibitors (such as PF-06873600) with CDK4/6 inhibitors and endocrine therapy have entered phase I/II clinical trials, showing promise in overcoming resistance to aromatase inhibitors and CDK4/6 inhibitors in ER + advanced breast cancer [362]. In the DNA damage response domain, CDK4/6 inhibitors such as palbociclib induce G1 phase arrest, driving tumor cells into S phase and amplifying gemcitabine-induced DNA damage to enhance its efficacy [363].

Notably, natural products demonstrate the potential to enhance synergistic effects in combination therapies and to reverse drug resistance, owing to their multi-target regulatory properties. For instance, ginsenoside Rg3 liposomes have shown a 15% improvement in progression-free survival in several phase II-III adjuvant trials for non-small cell lung cancer and hepatocellular carcinoma by targeting both the tumor microenvironment and cancer cells [365]. However, they are still not approved for use as monotherapies. Curcumin combined with kinase inhibitors like sorafenib modulates metabolism and the tumor microenvironment, enhancing antitumor effects [366]. Resveratrol and quercetin combined with CDK inhibitors activate the p53 pathway, overcoming resistance caused by Rb loss and achieving an objective response rate of 65% in colon cancer models [368]. Furthermore, isothiocyanate compounds like sulforaphane, when combined with PARP inhibitors, enhance synthetic lethality by amplifying DNA damage, significantly reducing recurrence risk in ovarian cancer [369]. These natural products often exert multi-target synergistic effects by modulating pathways such as nuclear factor kappa-light-chain-enhancer of activated B cells and PI3K/AKT. However, despite being natural, these products are not without risk. Potential adverse reactions, particularly hepatotoxicity, necessitate rigorous scientific evaluation, standardization, and careful management during clinical translation to ensure patient safety.

## Conclusions and perspectives

The systemic dysregulation of the cell cycle, often through the accumulation of genetic mutations, is a hallmark of nearly all cancers, regardless of the origins of the tumors. This leads to uncontrolled cell proliferation and genomic instability [335]. Notably, distinct tumor types (such as cervical cancer [378], endometrial cancer [379, 380], prostate cancer [381], colorectal cancer [382, 383], oral cancer [384, 385], liver cancer [386], gastric carcinoma [387–389], pancreatic cancer [390, 391], esophageal cancer [392, 393], lung cancer [394], and breast cancer [395]) exhibit marked heterogeneity in the nature and extent of cell cycle abnormalities (Table 3). Such heterogeneity not only poses a major challenge to uniform therapeutic strategies but also reflects the intrinsic complexity and evolutionary adaptability of tumors. Consequently, the systematic analysis of the dynamic molecular mechanisms that regulate tumor cell cycles has been recognized as a crucial undertaking in both fundamental research and clinical translation, serving as a vital route to overcoming this complex disease.

**Table 3:** Changes in cell cycle of several common cancers

Phase					
Cancer	G0/G1	G1/S	S/G2	G2/M	References
Cervical cancer					[378]
Endometrial cancer					[379, 380]
Prostate cancer					[381]
Colorectal carcinoma					[382, 383]
Oral cancer					[384, 385]
Liver cancer					[386]
Gastric carcinoma					[387–389]
Pancreatic cancer					[390, 391]
Esophageal cancer					[392, 393]
Lung cancer					[394]
Breast cancer					[395]

denotes promotion,  denotes blocking,  denotes no change

This review reveals the profound complexity and high interconnectedness of tumor cell cycle regulatory networks. Their dysregulation constitutes a dynamic system driven by multifaceted mechanisms and goes far beyond the simple activation or inactivation of the “core engine” the Cyclin-CDK-CKI pathway. The tumor cell cycle is modulated by the transcription-epigenetics-non-coding RNA axis, which reshapes the expression patterns of cell cycle-related genes and the PTMs, which act as molecular switches, precisely regulating the activity, stability, and localization of key proteins. Cell cycle checkpoints, which function as “quality control gates”, maintain genomic integrity during DNA damage, replication stress, or abnormal chromosome separation. Simultaneously, metabolic reprogramming and signaling pathways, deeply embedded within the tumor microenvironment, further amplify malignant phenotypes through feedback loops. These modules demonstrate tumor heterogeneity and therapeutic resistance mechanisms, and their dynamic crosstalk highlights the clinical significance of several biomarkers in diagnosis, prognostic evaluation, and treatment decision-making, including Cyclin D1, p27 subcellular localization, telomere length, and CIN/MSI status.

Currently, cell cycle-targeted therapeutic strategies mainly encompass direct CDK inhibition, synthetic lethality, and combination therapies. These strategies have demonstrated clinical benefit in selected tumor types and, to some extent, have expanded the therapeutic window through the exploitation of the multi-target properties of natural compounds. However, significant challenges remain, including regulatory network redundancy, tumor genetic heterogeneity, off-target toxicity, and the rapid emergence of compensatory signaling pathways. Accordingly, future research in tumor cell cycle regulation must move beyond single-target paradigms toward a systems-level framework that emphasizes multidimensional network modulation.

Several directions merit particular attention. First, integrating multi-dimensional data is essential to decipher the dynamic rewiring of cell cycle control and to target cancer cell phenotypic plasticity, with the long-term goal of transforming aggressive malignancies into manageable chronic diseases. Second, within highly heterogeneous tumor microenvironments, the development of dynamic, multi-omics-based biomarkers that reflect the functional state of the cell cycle will be crucial for optimizing therapeutic timing, monitoring treatment response, and anticipating resistance. Third, emerging technologies should be leveraged to explore innovative strategies that induce irreversible cell cycle exit in cancer cells while minimizing genomic instability and pro-inflammatory microenvironmental effects. Fourth, exploiting non-oncogene dependencies at specific cell cycle checkpoints offers opportunities to design highly selective synthetic lethal therapies that may preserve cell cycle homeostasis in normal tissues. Finally, given the intrinsic multi-target pharmacological properties of natural products, the integration of nanodelivery systems, structural optimization, and rational combination strategies may effectively overcome resistance mechanisms while improving pharmacokinetics and expanding therapeutic windows.

Overall, the thorough investigation of the dynamic regulation of tumor cell cycles is essential to understanding their pathogenesis. This research serves as the primary catalyst for converting basic science into therapeutic interventions. Only through systematic exploration across multiple scales and dimensions can we achieve effective interventions in cell cycle processes. This approach enhances treatment precision, delays the onset of drug resistance, improves patient outcomes, and ultimately provides a robust scientific foundation and innovative pathways for conquering cancer.

## Data Availability

Not applicable.
